# Machine learning approaches to dietary classification from dental microtexture in primates

**DOI:** 10.1038/s41598-026-47350-8

**Published:** 2026-04-28

**Authors:** Ferran Estebaranz-Sánchez, Kristina Kit, Juan José Ibáñez Estevez, David R. Insua, Simón Rodríguez Santana, Laura M. Martínez

**Affiliations:** 1 Cultural Landscape Research Group, IMF-CSIC, Egipcíaques 15, 08001 Barcelona, Spain; 2 DataLab, Institute of Mathematical Sciences, ICMAT-CSIC, 28049 Madrid, Spain; 3 ICAI Engineering School, Universidad Pontificia de Comillas - IIT, 28015 Madrid, Spain; 4 Departament de Biologia Evolutiva, Ecologia i CC Ambientals, Facultat de Biologia, Universitat de Barcelona, 08028 Barcelona, Spain; 5 Institut d’Arqueologia de la Universitat de Barcelona, Montalegre 8, 08001 Barcelona, Spain

**Keywords:** Computational biology and bioinformatics, Ecology, Ecology, Evolution, Zoology

## Abstract

Dental microwear texture (DMT) analysis is a critical proxy for reconstructing the diets of extant and extinct mammals. While craniodental morphology reflects selective pressures across evolutionary timescales, microwear captures localized, short-term dietary signals over weeks to months. However, the high dimensionality of modern 3D surface microtexture data, often spanning disparate parameter sets (such as ISO standards and scale-sensitive fractal analysis, SSFA), complicates classification, particularly when working with limited paleontological datasets. To address this, we present a robust machine learning pipeline designed to automatically classify primate samples ($N = 99$) across 6 dietary groups and 7 species. Our methodology leverages a nested leave-one-out cross-validation framework to evaluate multiple classifiers, including multinomial logistic regression (MLR) with various regularization approaches, Naive Bayes, and tree-based ensemble algorithms (e.g., Random forests, XGBoost). Our results demonstrate that Lasso-regularized MLR and Naive Bayes yield the highest predictive performance while enforcing strict feature selection to maintain interpretability. Crucially, models relying exclusively on ISO parameters consistently outperformed those using SSFA, as ISO variables better capture the microroughness localized mechanical abrasions generated by specific diets in our dataset. Furthermore, the integration of novel Fourier-based descriptors and isotropy variables significantly enhanced models’ discriminating power. By providing a mathematically rigorous framework to isolate precise ecological signals from noisy, high-dimensional data, this approach enables more accurate and reproducible dietary classifications. Ultimately, refining these dietary reconstructions is essential for resolving broader questions regarding niche partitioning, species evolution, and paleoecological dynamics.

## Introduction

The study of diet and feeding habits of primates, as well as other mammals, is crucial to understand their ecology and evolution^1–4^. Dietary reconstruction is based on the analysis of different proxies tested in extant species with well-known dietary habits^5^. The selection of such proxies is far from trivial as they record diet across different temporal scales and can, therefore, provide contradictory palaeodietary information, resulting in different reconstructions^5,6^. In this context, proxies based on the analysis of craniodental morphology provide information related to evolutionary processes (*e.g.* adaptation, speciation). In contrast, other proxies, such as 2D microwear (MW) or 3D microtexture (DMT) patterns, provide finer (palaeo)dietary information at seasonal or, even, weekly resolution^7–9^. This may explain, for example, the apparent contradiction between the craniodental morphology of some fossil species and their microwear data, *e.g.* in *Australopithecus anamensis*^10,11^ or in *Paranthropus* genus^12,13^. Dental microwear and microtexture have thus become important proxies^5,14–18^ to infer the dietary habits of extant^19–21^ and extinct primates and hominins^10–13,15^ since they provide useful information within a relatively short time frame before the death of the individual, while it is a technique that can be effectively applied to fossils with no chronological limit.

Microwear/microtexture studies have different objectives, including (1): *Within-group variability analysis* (*e.g.* intraspecific, intrapopulation). These studies assess variations within a group (*e.g.* population, species), to identify how factors such as sex^22–24^, age^21,22,24–27^, chronology or location^10,12,24,28^ may influence microwear and microtexture patterns. This approach is particularly useful when the aim is to characterize internal variability without necessarily reconstructing a specific diet; (2) *Between-group variability of unknown diet groups* (*e.g.* interspecific, interpopulation, intergroup): This category includes comparisons among several groups whose diets are not well established. The objective is to identify relative differences in abrasion or texture between groups. This approach highlights ecological contrast (abrasive vs softer diets) between groups, even in the absence of a known dietary reference sample^29–32^; (3) *Between-group variability of known diet groups* to determine whether microwear or microtexture analysis can significantly identify groups (*e.g.* populations or species) whose diets are known. For example, microwear and microtexture have been shown to differentiate various groups of recent hunter-gatherers and agriculturalists^20,21^, as well as cecopithecoid taxa with distinct feeding regimes^16^. This approach is crucial for building reference datasets, wich subsequently underpin paleodietary interpretations. Reference micrower models both in Hominoidea and Cercopithecoidea^19,33,34^ were developed using this strategy and have since been applied to the study of fossil hominins and other primates^10–13,17,35^; (4)* Reconstruction of the palaeodiet of a group *(*e.g.* species, population) This approach directly compares both signatures of a fossil species or population with an appropriate reference sample of know diet. By evaluating their similarity researchers can infer the dietary regime of extinct individuals. This method is widely applied in palaeoanthropology and bioarchaeology and has proven effective in reconstructing the diets of fossil hominins, non-human primates, and past human populations^11,15,17,22,27,36–38^.

Numerous articles address more than one of the previously mentioned objectives, including intra and intergroup analysis of unknown dietary groups^39,40^, between-group variability of known diet groups and paleodietary reconstruction of a specific archaeological or paleontological group^36,41^, intraspecific analysis, reconstruction of the paleodiet of a specific group^10^ or test the relation between microwear and isotopic signal^6,17^.

Systematic approaches based on statistical/machine learning techniques have been used in microwear/DMT studies, with the choice of the methodologies dependent on the number of variables. The buccal microwear pattern consists of 15 variables^22,39^, while occlusal (high-magnification) include fewer variables, typically between 3 and 6^42–47^. Therefore, the statistical strategy when analyzing buccal microwear patterns usually emphasizes reducing data dimensionality to represent the groups studied graphically and analyze their similarity. One of the most commonly used methods is Principal Component Analysis (PCA)^20,26,39,48^, which projects the data in directions resulting from the linear combination of the original variables^49^. This facilitates 2D plot representations of the original data in the directions of maximal information^50^. Alternatively, other studies have chosen Discriminant Analysis (DA)^10,11,13,26^ or Canonical Variate Analysis^15,31,51^, which maximally separates the predefined groups of interest based on the data. In general, dimensionality reduction techniques are not typically used in occlusal microwear, at least for primate and hominin studies, the exception being^17^ with *Theropithecus oswaldi*. However, in low-magnification occlusal studies with a similar number of variables, multidimensionality reduction techniques are commonly applied^52–56^. Ultimately, primate and hominin high-magnification occlusal microwear pattern variability is represented through simple biplots of pitting incidence vs. scratch width^42,46,47^, pitting incidence vs. pit width^43^ or number of scratches vs. number of pits^45^.

The statistical/Machine Learning (ML) strategy for DMT studies also depends on the set of variables used (typically, scale-sensitive fractal analysis (SSFA) and dental surface texture analysis from the international standard (ISO 25178)) as each set size may vary among studies. Broadly, ML offers a data-driven framework capable of automatically identifying complex, high-dimensional patterns to classify specimens based on these multivariate textural signatures. Primate and hominin SSFA studies, conducted on both occlusal and buccal surfaces, typically rely on a limited number of parameters, most commonly between two and six^14,57–64^. With rare exceptions^58^, multidimensionality reduction techniques are generally not applied in SSFA analyses; instead, palaeodietary differences are usually explored using bivariate plots based on the most discriminant variables, most often epLsar (Length-scale Anisotropy of the relief) versus Asfc (Area-Scale Fractal Complexity)^14,60,63^. In contrast to SSFA studies, few ISO-based DMT articles in primates and hominins have been published to date, either in the occlusal^65–67^ and the buccal surfaces^16,17^. Nevertheless, the analysis of 3DST involves the use of a large number of ISO 25178 / ISO 12781 parameters^16,17,68^, so that the use of dimensionality reduction techniques is more common^68^, especially PCA^69–73^, as well as DA^69,70^.

With respect to selecting the most relevant parameters in 3DST studies, a pioneering study on occlusal 3DST in primates^74^ highlighted a subset of ISO parameters (S5v, Sq, Vm, Spd, Sha, Sda) as indicative of the interaction between food and enamel during mastication. However, only a limited number of variables were considered. Later studies expanded the set of ISO variables, increasing analytical complexity and leading to what has been called a “jungle of parameters”^75^. Discriminative power varies across studies and sample compositions, likely due to high inter-variable correlations^16,17,68^. Moreover, the criteria for defining a parameter as diet-discriminative differ between contributions^75^ due to multiple factors (*e.g.* data sources, noise contamination and others). Thus, no universal set of optimal discriminative parameters has emerged. Still, some trends suggest that combining spatial and height parameters yields better discrimination power^76^.

Our study develops a methodological pipeline for preprocessing and analysing DMT variables (3DST –ISO– or SSFA) alongside Fourier and Furrows variables (labeled Other) for palaeodietary reconstruction by performing groups and species classification, with a focus on model interpretability and the assessment of variable relevance^77^. Furrow and isotropy parameters have been used recently to discriminate dietary regimes of mammals^65,67,78^. Specifically, we aim to contribute by (i) *proposing a framework* for identifying effective statistical and machine learning methods to classify primate species using buccal 3DST data, prioritising interpretability while incorporating ensemble approaches (*e.g.*, random forests, boosting) for contrast; (ii) providing a reusable *code pipeline* for identifying key SSFA and ISO variables, facilitating future comparisons and data interpretation; and (iii) evaluating *variable importance* obtained in the constructed classification models to determine which features best discriminate microtexture patterns.

## Results

### Data preprocessing results

**Fig. 1:**
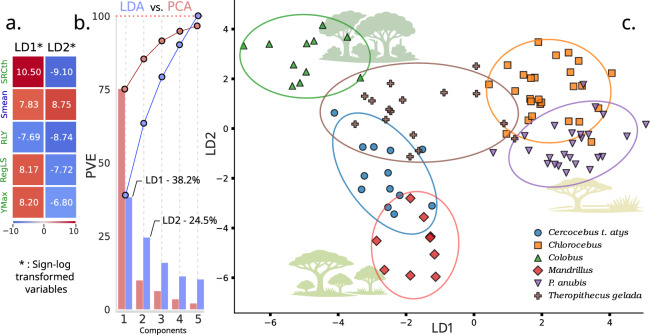
(*Left* - **a**) First 5 variables of the main two LDA components for group classification in preprocessed dataset. Values displayed in log-scale, with signs re-introduced afterwards. Variable names are color-coded, ISO in blue, SSFA in green. (*Center* - **b**) Proportion of variance explained (PVE) for both LDA (in blue) and PCA (in red) for the dataset. (*Right* - **c**) Data projection using the first two LD directions. Ellipses show Gaussian fits covering $2\sigma$ for each label. Best viewed in color.

We applied the full data preprocessing pipeline to maximise the information available per group and species. For complete information on the dataset, including final variables selected, please refer to “Data preprocessing” Section. Despite the strong non-normality and internal collinearity observed within several variable sets (see “Methods” Section), most variables still displayed group and species-level structure when projected into low-dimensional space. The LDA visualization in Fig. 1 (*right*) shows that, after preprocessing, LD directions contain a meaningful discriminative signal that distinguishes groups in this reduced dimensionality space. This motivated retaining all variables, rather than restricting the analysis to the subset passing normality or *variance inflation factor* (VIF^79^) thresholds, since the full feature representation provides more informative class separation.

In the left panel (**a**) of Fig. 1 we display top-5 variable loadings of the first two LD components (in sign-log-transformed scale). To compare to the usual decomposition using PCA, in the center panel (**b**) we show the proportion of variance explained (PVE) by the addition of each LDA (blue) and PCA (red) component. Even though PCA explains a larger portion of the variance in the initial components, since the LDA projections are constructed to maximize separability under the LDA assumptions^50^ we see that the data clusters appear quite separate in the low-dimensionality figure, included in the right panel of the figure (**c**), including the corresponding Gaussian ellipses to illustrate group dispersion. This LDA projection reveals biologically interpretable structure: LD1 separates folivorous taxa such as *Colobus* (negative values) from open-habitat feeders like *Chlorocebus* and *P. anubis* (positive values), while *Theropithecus gelada* spans a broader range along this axis. LD2 differentiates *Mandrillus* and *Cercocebus atys*, both associated with hard or brittle dietary components, from the remaining groups, consistent with *Cercocebus* specializing in hard fruits in closed habitats and *Mandrillus* exploiting a diverse diet including fruits, leaves, and brittle seeds.

### Classification models

We report performances of all models for the group-classification task in Table 1 (for the species classification results, see the SI). Rows correspond to the four variable sets (ISO, SSFA, Other –Fourier and Furrows– and All). For each method, we display the best result obtained from either the VIF-filtered data or its non-filtered counterpart, so reported results in Table 1 include only the best one between them. We also evaluated each method using the original and standardized datasets, although these generally provided worse results than those presented here. For each metric, the highest-performing method is highlighted in bold red and the second highest in bold, with ties indicated when applicable. Given the small multiclass dataset (6 groups, 7 species), performance was estimated using LOOCV, and errors represent the standard errors of macro-averaged metrics. Micro-averaged accuracies are denoted $\upmu$-*Acc.*, macro-averaged as *M-Acc.*. We also include $\text {LDA}_C$ (LDA applied to the VIF-filtered datasets) for comparison^80^. For a complete definition of all models and metrics, please see “Modeling and evaluation” Section.

We benchmarked results against two baselines: (i) random classifiers yield accuracies near 0.16 ($\approx 1/6$) for groups and 0.17 ($\approx 1/7$) for species; (ii) LDA, the standard in species classification, was applied after preprocessing, showing improved performance. Table 1 shows that VIF colinearity removal generally enhances accuracy in LDA, except for Other, where variable reduction may amplify noise. QDA did not outperform LDA, consistent with small, noisy datasets where LDA’s lower variance offers greater robustness^81^.

**Table 1:** Classification results for Groups.

	**Metrics**	LDA	$\hbox {LDA}_C$	QDA	MLR	Lasso	EN	NB	SVM	RF	XGB	NGB
	$\upmu$-Acc.	0.27	0.28	0.21	0.33	0.32	0.33	***0.34***	0.30	0.28	0.32	***0.34***
	*M*-Acc.	$0.26 \pm 0.03$	$0.24 \pm 0.06$	$0.17 \pm 0.07$	**0.32±0.04**	$0.31 \pm 0.05$	$0.31 \pm 0.06$	***0.38 ± 0.10***	$0.24 \pm 0.09$	$0.23 \pm 0.07$	$0.29 \pm 0.06$	$0.31 \pm 0.05$
I	Prec.	$0.25 \pm 0.04$	$0.26 \pm 0.05$	$0.18 \pm 0.06$	$0.35 \pm 0.07$	$0.34 \pm 0.07$	$0.33 \pm 0.05$	***0.38 ± 0.07***	$0.23 \pm 0.08$	$0.23 \pm 0.06$	$0.29 \pm 0.06$	**0.37 ± 0.05**
S	Rec.	$0.26 \pm 0.03$	$0.24 \pm 0.06$	$0.17 \pm 0.07$	**0.32±0.04**	$0.31 \pm 0.05$	$0.31 \pm 0.06$	***0.38 ± 0.10***	$0.24 \pm 0.09$	$0.23 \pm 0.07$	$0.29 \pm 0.06$	$0.31 \pm 0.05$
O	*F*1	$0.26 \pm 0.03$	$0.24 \pm 0.05$	$0.16 \pm 0.06$	***0.33 ± 0.05***	**0.32 ± 0.05**	**0.32 ± 0.05**	**0.32 ± 0.05**	$0.22 \pm 0.08$	$0.23 \pm 0.06$	$0.29 \pm 0.06$	**0.32 ± 0.03**
	C-$\kappa$	0.11	0.10	0.04	**0.17**	0.15	0.16	***0.22***	0.11	0.09	0.16	**0.17**
	AUC-ROC	0.60	0.67	0.51	**0.71**	**0.71**	***0.73***	0.68	**0.71**	0.67	0.69	0.61
	$\upmu$-Acc.	0.19	0.28	0.23	**0.31**	**0.31**	0.29	0.27	0.30	***0.34***	0.30	0.30
	*M*-Acc.	$0.15 \pm 0.05$	$0.22 \pm 0.08$	$0.15 \pm 0.10$	$0.25 \pm 0.08$	**0.26 ± 0.06**	$0.22 \pm 0.08$	***0.27 ± 0.03***	$0.22 \pm 0.10$	$0.25 \pm 0.10$	$0.22 \pm 0.09$	$0.23 \pm 0.08$
S	Prec.	$0.14 \pm 0.05$	$0.21 \pm 0.07$	$0.08 \pm 0.05$	$0.24 \pm 0.06$	***0.27 ± 0.05***	$0.22 \pm 0.06$	***0.27 ± 0.04***	$0.17 \pm 0.08$	$0.23 \pm 0.07$	$0.21 \pm 0.08$	$0.26 \pm 0.07$
S	Rec.	$0.15 \pm 0.05$	$0.22 \pm 0.08$	$0.15 \pm 0.10$	$0.25 \pm 0.08$	**0.26 ± 0.06**	$0.22 \pm 0.08$	***0.27 ± 0.03***	$0.22 \pm 0.10$	$0.25 \pm 0.10$	$0.22 \pm 0.09$	$0.23 \pm 0.08$
F	*F*1	$0.15 \pm 0.05$	$0.21 \pm 0.08$	$0.10 \pm 0.06$	$0.24 \pm 0.07$	***0.26 ± 0.06***	$0.22 \pm 0.07$	***0.26 ± 0.03***	$0.19 \pm 0.08$	$0.24 \pm 0.08$	$0.21 \pm 0.08$	$0.23 \pm 0.06$
A	C-$\kappa$	$-0.01$	0.09	$-0.04$	**0.13**	**0.13**	0.10	0.11	0.09	***0.16***	0.11	0.10
	AUC-ROC	0.57	0.64	0.49	0.65	0.67	***0.68***	0.62	0.65	***0.68***	0.64	0.57
	$\upmu$-Acc.	***0.36***	0.30	0.22	0.33	0.34	0.32	0.23	0.35	0.26	0.24	***0.36***
O	*M*-Acc.	***0.30 ± 0.07***	$0.23 \pm 0.10$	$0.16 \pm 0.07$	$0.26 \pm 0.09$	**0.28 ± 0.07**	$0.24 \pm 0.09$	$0.18 \pm 0.07$	$0.24 \pm 0.13$	$0.21 \pm 0.07$	$0.17 \pm 0.08$	$0.26 \pm 0.11$
t	Prec.	***0.34 ± 0.05***	$0.22 \pm 0.08$	$0.14 \pm 0.05$	$0.25 \pm 0.08$	**0.31 ± 0.05**	$0.24 \pm 0.07$	$0.18 \pm 0.07$	$0.16 \pm 0.08$	$0.23 \pm 0.06$	$0.13 \pm 0.06$	$0.25 \pm 0.08$
h	Rec.	***0.30 ± 0.07***	$0.23 \pm 0.10$	$0.16 \pm 0.07$	$0.26 \pm 0.09$	**0.28 ± 0.07**	$0.24 \pm 0.09$	$0.18 \pm 0.07$	$0.24 \pm 0.13$	$0.21 \pm 0.07$	$0.17 \pm 0.08$	$0.26 \pm 0.11$
e	*F*1	***0.31 ± 0.06***	$0.22 \pm 0.08$	$0.15 \pm 0.06$	$0.25 \pm 0.08$	**0.28 ± 0.06**	$0.23 \pm 0.08$	$0.18 \pm 0.07$	$0.18 \pm 0.09$	$0.21 \pm 0.06$	$0.15 \pm 0.07$	$0.24 \pm 0.09$
r	C-$\kappa$	***0.20***	0.12	0.01	0.16	0.17	0.14	0.06	0.14	0.07	0.02	**0.18**
	AUC-ROC	**0.67**	**0.67**	0.53	***0.68***	**0.67**	**0.67**	0.64	0.65	0.62	0.54	0.62
	$\upmu$-Acc.	0.22	0.32	0.11	**0.34**	***0.36***	0.31	0.32	0.16	0.27	0.27	0.31
	*M*-Acc.	$0.18 \pm 0.06$	$0.28 \pm 0.05$	$0.10 \pm 0.02$	$0.32 \pm 0.06$	**0.34 ± 0.06**	$0.27 \pm 0.08$	***0.35 ± 0.07***	$0.11 \pm 0.07$	$0.23 \pm 0.07$	$0.23 \pm 0.06$	$0.28 \pm 0.04$
A	Prec.	$0.19 \pm 0.07$	$0.28 \pm 0.05$	$0.12 \pm 0.04$	**0.32 ± 0.04**	***0.34 ± 0.04***	$0.27 \pm 0.08$	$0.31 \pm 0.06$	$0.05 \pm 0.03$	$0.23 \pm 0.06$	$0.23 \pm 0.05$	$0.30 \pm 0.03$
l	Rec.	$0.18 \pm 0.06$	$0.28 \pm 0.05$	$0.10 \pm 0.02$	$0.32 \pm 0.06$	**0.34 ± 0.06**	$0.27 \pm 0.08$	***0.35 ± 0.07***	$0.11 \pm 0.07$	$0.23 \pm 0.07$	$0.23 \pm 0.06$	$0.28 \pm 0.04$
l	*F*1	$0.18 \pm 0.07$	$0.28 \pm 0.05$	$0.10 \pm 0.02$	**0.31 ± 0.05**	***0.33 ± 0.05***	$0.27 \pm 0.07$	$0.30 \pm 0.05$	$0.07 \pm 0.04$	$0.23 \pm 0.06$	$0.23 \pm 0.05$	$0.29 \pm 0.03$
	C-$\kappa$	0.06	0.15	$-0.08$	0.18	***0.20***	0.13	**0.19**	$-0.13$	0.08	0.08	0.13
	AUC-ROC	0.50	0.67	0.46	0.68	0.68	***0.72***	0.67	0.28	**0.71**	0.67	0.63

Each model tuned for optimal performance per variable set with (i) the *original*, preprocessed data without the *variance inflation factor* (VIF) filter for collinearity, and (ii) the *clean* (with _*C*_ subscript for LDA), VIF-filtered and no collinear variables. Best performance in each case is highlighted in Bolditalic, second-best in bold only. For full description on each method (columns), and metric (rows) please refer to “Modeling and evaluation” Section.

It is worth noting that recent preceding publications relied primarily on LDA^80^, which, even when aided by our improved preprocessing pipeline, still performs worse overall than most of the classifiers evaluated here (except for the Other variable set). This leads us to believe that LDA should only be used as a reference point for datasets similar to ours, and that other methods should be used for prediction on new samples. Note also that our evaluation here is intentionally stringent. By using LOOCV and reporting both macro- and micro-averaged metrics, we adopt a conservative scoring strategy that minimizes optimistic bias and class-imbalance effects. As a result, the reported metric values may appear modest, which is expected for a 6-class classification problem with fewer than 100 observations evaluated under such strict conditions. In this setting, each fold tests a single left-out individual, leading to particularly harsh penalties across all performance metrics.

**Fig. 2:**
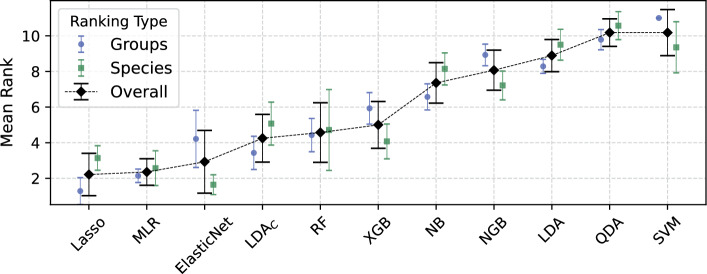
Mean ranking across metrics for all methods for groups (blue dots), species (green squares) and overall (black diamonds). Lower is better. Best viewed in color.

To further illustrate these results, we ranked all models in “Modeling and evaluation” Section from best to worst for each dataset and metric in both the group- and species-level classification tasks. We then computed the mean rank and its standard error for each method across all metrics and datasets, as shown in Fig. 2 (lower values indicate better performance). MLR^50^ and its regularized variants (Lasso and Elastic Net) consistently ranked highest, reflecting their ability to handle multiclass problems with collinearity and noise through coefficient shrinkage. NB was competitive for ISO variables on non-VIF-filtered data, often leading on the original datasets, which suggests that VIF filtering may remove variables carrying independent predictive signal important for NB. LDA performed best with the Other variable set, benefitting from VIF filtering ($\hbox {LDA}_C$ ranking 4th), consistent with its reliance on linear decision boundaries and low-variance estimation (for additional details see SI).

MLR models performed similarly with ISO variables alone and the full All set, suggesting that the inclusion of additional variables yields only marginal gains. Overall, ISO variables provided the strongest and most stable results, whereas SSFA and Other did not show a clear performance benefit. Consequently, ISO variables alone emerge as the most robust choice in this setting, supporting the majority of top-performing metrics in both classification tasks (see SI for further details and species-level results). Although these findings are not necessarily generalizable, they motivate further investigation using larger and more diverse datasets.

### Automated variable selection

**Fig. 3:**
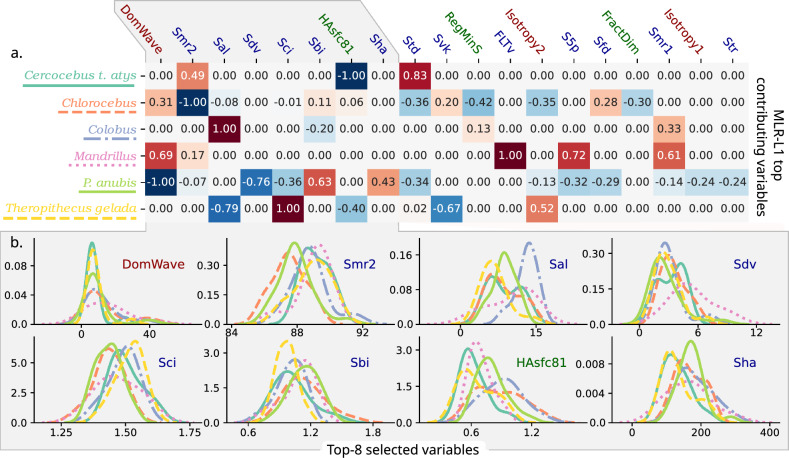
*Top* (**a**): Highest 19 Lasso MLR coefficients for group classification re-scaled group-wise. Variable names are color-coded, with ISO variables in blue, SSFA, in green, and Other, in red. *Bottom* (**b**): KDE plots for the top 8 variables. Groups are distinguished with colors and line types, reflected in the names and underlines for each group in the top-part of the figure. Best viewed in color.

Among all the methods included in our pipeline, MLR models seem to best suit our interests since they provide improved robustness and clear interpretation, with their regularized variants (*Lasso*, *Elastic Net*) being particularly apt to perform automated variable selection while maintaining high performance (see Table 1). The results in Fig. 3 here correspond to Lasso MLR applied to the All dataset (73 variables across ISO, SSFA, and Other) without VIF filtering so that Lasso can select variables directly. Group-level results are presented here, while species-level results are included in the SI.

After training on the classification task, Lasso reduces the number of used variables from 73 to 32 for both group and species classification (for more details on the model’s properties, please refer to “Modeling and evaluation” Section). The selected sets are very similar: FLTv and Spk (groups) are replaced by S5v and Sz (species), all of which play minor roles. This stability is expected because species classification refines groups by subdividing one of them. Interpretation of these results is aided by the Lasso coefficients (Fig. 3, top). Variables are ordered by importance (left = highest), and the sign of the re-scaled coefficients reflects whether higher values make more or less likely belonging to a given group.

The kernel density estimates (KDE^50^) plots for each figure (Fig. 3, bottom) help understand these choices by the model. For example, high Sal (an ISO autocorrelation measure) is characteristic of *Colobus* and aligns with its positive coefficients, whereas *Chlorocebus* and *T. gelada* show lower Sal. *T. gelada* also shows high Isotropy2 and Sci but low surface complexity. *Mandrillus* and *Cercocebus*, which process brittle foods, are distinctly non-isotropic, with high Smr1, S5p, and Smr2. *Papio* surfaces tend to be rounded and non-isotropic. Across models, variables such as DomWave, Smr2, Sal, Sci, and HAsfc81 consistently rank among the most informative features.

## Discussion

### Reducing the number of parameters

Microtexture datasets are typically high-dimensional, especially when large ISO parameter sets are used, as in many recent buccal and occlusal studies^9,16,17,82–84^. This dimensionality issue increases redundancy and complicates interpretation, underscoring the need for explicit variable selection. In contrast, SSFA relies on fewer descriptors and generally produces lower-dimensional representations (“Introduction” Section). In our dataset, correlation analyses showed that variables within both frameworks were either weakly related or strongly collinear, as showcased in Fig. 5. Applying a collinearity filter (“Data preprocessing results” Section) reduced the ISO set to nine variables (Sku, Str, Std, Shv, Smr1, Sfd, Sci, Smean, Stdi), retaining complementary height, spatial, and volume descriptors as recommended previously^76^. These parameters have demonstrated sensitivity to buccal microtexture variation^16,69,85,86^.

After filtering, ISO variables accounted for nearly half of the retained predictors and yielded the strongest classification performance across metrics (Table 1). This indicates that, once redundancy is controlled, ISO descriptors capture most of the discriminative signal in our sample. Fourier-based variables (the Other set), such as isotropy and dominant wavelength, also contributed to group separation, as shown by the Lasso coefficients (Fig. 3).

Although not the primary focus here, SSFA variables showed lower internal collinearity than ISO parameters, consistent with previous work^87^. Their more independent structure suggests that they may complement ISO descriptors, even if they contributed less to discrimination in the present analysis.

### Specific versus generic level comparisons

Our study applied several statistical tests to characterize cercopithecid species of known diet to suggest key statistical/ML tools to classify and discriminate species based on microtexture patterns. One relevant point to comment is that whether we consider only the ISO variables, the SSFA ones or both together, the performance of classifiers is better when working at the more genus (group) level due to there being one fewer class (with *Chlorocebus pygerithrus* and *Chlorocebus aethiops* grouped in a single genus category *Chlorocebus sp*) than at the species level (*i.e.*, when both *Chlorocebus* species are treated independently; see SI with the species level analysis). In studies of occlusal SSFA, whether working at the genus or species level as a factor or grouping variable, significant differences were observed in extant primates (^34^); however, the authors suggested that in the absence of substantial differences between species of the same genus, the most pertinent analysis would be a generic-level comparison. A previous study^16^ found that there were no significant statistical differences between both *Chlrococebus* species, with only the (Sal) variable showing a significant difference out of 29 of them. Our results here support that working at the genus level performs better than at the species level. This approach is particularly relevant in studies of fossil primates^88^, where sampl sizes are often limited. In such contexts, combining closely related taxa may help increase statistical power^34,88^ ; however, this should only be done when there is reasonable evidence that the taxa included share a comparable dietary range. Therefore, grouping specimens at the genus level should not be considered a purely taxonomic decision but rather an analytical strategy that assumes similar dietary behaviour among the taxa included. Under these conditions, increasing the effective sample size may improve the robustneess of paleodietary interpretations^88,89^.

### Comparison of different classifiers and types of variables 

Another objective of this study was to assess the relative performance of different classifiers. For models using only ISO variables, the best results across most metrics were obtained with the NB classifier, followed by MLR and, less consistently, NGB. Although NB achieved the highest performance when restricted to ISO variables, our final analyses rely on Lasso-regularized MLR, as this model incorporates all variable sets simultaneously (fourth row in Table 1) and yields the best overall performance. Moreover, MLR models consistently ranked among the top-performing methods across metrics (Fig. 2), a property that is particularly valuable in applied contexts where stability and interpretability across variable configurations are preferred over isolated performance peaks.

LDA was included to maintain comparability with previous analyses of the same sample^16,17^. Despite the proposed preprocessing pipeline, however, LDA consistently underperformed relative to most other classifiers. This likely reflects the mismatch between LDA’s assumptions (*e.g.*, class structure and covariance homogeneity) and the characteristics of our dataset, as well as the limited sample size per class, which makes the method sensitive to small variations even under LOO cross-validation. Performance improved after VIF filtering ($\hbox {LDA}_C$), but remained inferior to NB and MLR across all metrics. Given these results, for this dataset (or others similar to it, particularly when using ISO variables), we suggest LDA should be considered a baseline reference rather than a competitive classifier, despite its traditional use in dental microtexture research. Given that many studies rely on DA or PCA for dimensionality reduction in similarly high-dimensional settings^71,78,85,86,90–94^, it is plausible that alternative classifiers would also yield improved performance in those contexts. In this regard, our results support a broader use of MLR, which has only rarely been applied in occlusal microwear studies^95,96^.

For models using SSFA variables, NB and Lasso MLR yielded the best results, with Lasso MLR demonstrating the most consistent performance. Uniquely, our analysis incorporated all 31 available SSFA parameters rather than the traditional subset of 2–4 variables (*e.g.*, Asfc, epLsar^87^). While this precludes direct generalization to past studies, it highlights the untapped potential of exploiting broader SSFA feature sets.

However, despite previous analyses have relied on SSFA for palaeodietary reconstruction^60,68,74,76,87^, our best overall classifiers relied exclusively on ISO parameters. This advantage likely arises because ISO variables capture microroughness, localized mechanical abrasions (*e.g.*, precise depths and volumes) tied directly to physical food-enamel interactions^74,97,98^. Conversely, SSFA measures broad, scale-invariant complexity, which may underestimate subtle geometric signatures required to differentiate closely related taxa. Finally, because ISO and SSFA describe the identical physical surface, combining them introduces severe multicollinearity and redundant noise rather than orthogonal discriminatory information, ultimately degrading the performance of sensitive models.

While combining ISO and SSFA variables might be expected to improve discrimination by providing complementary information^74,99^, our results do not support this assumption. Although previous studies have included both variable types, they have generally analysed them separately^74,83,84,99–101^, and only a few have combined them within a single statistical framework^9,71,80,93,102^. To our knowledge, none have systematically compared their joint discriminative power across taxa with different diets^68^. In this study, combining ISO and SSFA variables resulted in similar or poorer performance than using ISO variables alone at both the group and species levels. By contrast, variables related to isotropy and dominant wavelength (Other set) contributed meaningful discriminatory information, and this was the only variable set for which LDA achieved its best overall performance (Table 1). Although such variables have been applied to other materials (*e.g.*, lithic tools^103,104^), they have not previously been incorporated into primate dental microtexture analyses. Therefore, we consider this finding warrants further investigation.

Beyond the choice of variables and algorithms, classification success varied notably depending on the specific ecology of the taxa analyzed (see the SI for further group-wise classification analysis). On the one hand, some groups are represented with fewer points than others, leading to variable statistical power for the different methods depending on each case. On the other hand, species possessing highly specialized and stereotyped diets naturally exhibit highly distinct and homogeneous microwear signatures, making them significantly easier for the algorithms to isolate in the feature space. In contrast, generalist or opportunistic species, whose diets fluctuate heavily with seasonal and regional availability, produce highly heterogeneous microtexture patterns. These broad dietary niches inherently result in overlapping statistical distributions in the feature space, thereby increasing the difficulty of algorithmic classification and driving down their specific accuracy metrics.

Finally, we emphasize that conclusions regarding variable selection must be interpreted cautiously given the limited sample size and the focus on buccal enamel surfaces. While ISO and SSFA variables may provide complementary information^74,105^, their combination within a single ML pipeline may introduce sufficient noise to offset potential gains. Consequently, trends identified here require validation using larger and more diverse samples, as well as occlusal surfaces, to assess whether (1) ISO variables consistently outperform SSFA in classification, (2) Fourier-based and furrow-related variables enhance ISO-based discrimination, and (3) ISO and SSFA parameters are better treated as complementary, but not directly combinable within the same analytical framework.

### Conclusions, limitations and future directions

Our findings indicate that, for buccal enamel surfaces in the cercopithecid sample analysed, ISO texture parameters provide greater discriminatory power than SSFA variables. Across all evaluated classifiers, models relying exclusively on ISO variables consistently achieved higher or more stable performance than those based on SSFA parameters or on the combined variable sets. This suggests that ISO parameters may be more sensitive to the microtexture differences relevant for distinguishing dietary groups in this specific sample, successfully capturing fundamental aspects of surface texture and the direct mechanical interaction between food particles.

Despite these promising results, several limitations must be acknowledged. First, the dataset is limited in size and this effect increases the influence of noise and individual-level variability, limiting the robustness of cross-validation estimates. The use of LOOCV allowed leveraging all available observations, but it also means that species with scarce representation may disproportionately affect classification results. Second, although our preprocessing pipeline reduced collinearity and improved the stability of the models, it cannot fully compensate for the inherent variability of microtexture data, nor for potential measurement noise introduced during scanning or data extraction. These factors could be improved upon by conducting similar studies in larger datasets.

These limitations directly inform several avenues for future research. Increasing sample size remains a priority, both to improve the stability of classification models and to enable more granular analyses (*e.g.*, seasonality or population-level variation). Additionally, the promising behaviour of variables related to isotropy and dominant wavelength (here included in the Other set) suggests that their role should be explored further across taxa and surface types. Beyond enlarging the dataset, more expressive statistical frameworks *e.g.*Bayesian hierarchical modelling or other probabilistic ML methods could substantially enhance inference by explicitly modelling uncertainty and allowing information to be shared across individuals, species, or dietary groups. This would potentially improve parameter interpretability and stabilize estimates in data-limited settings. Such approaches may also help quantify the degree to which variability arises from individual- versus group-level factors, which is difficult to assess with classical methods. Further methodological work is needed to evaluate how these frameworks respond to different variable types, particularly in studies where dimensionality reduction is common. Finally, replicating this analysis on occlusal enamel surfaces, which differ functionally from buccal ones, would be crucial to determining whether the relative advantage of ISO variables generalizes beyond the current context.

Taken together, our study offers a comprehensive and reproducible pipeline for the preprocessing, selection, and statistical classification of microwear-derived dietary data. By systematically comparing a broad set of interpretable classification models, our framework clarifies which variables and variable sets contribute most strongly to separating dietary groups, providing a robust methodological foundation that can be readily extended to new datasets and to future applications in both extant and fossil dietary reconstruction.

## Methods

### Primate reference collection

This study is based on the open-access dataset of^16^, which comprises 98 lower second molars (LM2) from seven African Cercopithecoidea species with distinct dietary adaptations (Table 2). Tooth selection follows the criteria described in^16^ and related studies^15,106^, with a single modification: *Chlorocebus pygerythrus* and *Chlorocebus aethiops* are grouped into a single category due to their similar habitats and diets. Results obtained under the original species-level separation are reported in the supplementary information. The sample sizes listed in Table 2 correspond to the raw dataset, whereas the effective number of observations may differ after preprocessing, as illustrated in Fig. 1. In particular, the application of the outlier filter may exclude some specimens when predefined criteria are met, resulting in smaller group sizes than those initially reported.

**Table 2:** Primate Cercopithecoidea sample and dietary information.

Species	Sample size	Region of origin	Habitat	Dietary category
*Colobus polykomus*	11	Liberia	Forest	Folive-seed eater
*Mandrillus sphinx*	9	Cameroon and Gabon	Forest	Folive-Frugivore-Seed eater
*Papio anubis*	25	Kenya	Savanna	Opportunistic omnivore
*Theropithecus gelada*	16	Ethiopia	Grasslands-highland plateaus	Grass eater
*Chlorocebus pygerithrus*	11	Tanzania	Savanna-woodland	Opportunistic omnivore
*Chlorocebus aethiops*	15	Kenya	Savanna	Opportunistic omnivore
* *Chlorocebus sp.*	26	East Africa	Savanna	Opportunistic omnivore
*Cercocebus atys*	13	Cameroon, Congo and Liberia	Forest	Hard object feeder

* = Category *Chlorocebus sp.* includes *Chlorocebus pygerithrus* and *Chlorocebus aethiops* individuals.

### Image processing

Buccal enamel surfaces were scanned using a Sensofar PluNeox confocal microscope at IMF-CSIC, with a $20\times$ objective (0.45 NA) following^16,17^. As required for buccal microwear^10,11,15^, teeth were positioned perpendicular to the light beam at a $90^\circ$ angle. Four non-overlapping $138\times 102 \upmu$ m sub-areas were selected from larger $650\times 550\upmu$ m fields. Surfaces were leveled using least squares, had form removed with a 2nd-degree polynomial^16^ and filtered (0.5% tail threshold and Gaussian $3\times 3$). For each subarea we extracted 45 ISO and 20 SSFA variables with SensoMAP 7 (Digital Surf) from which we used a subset of 31 (further details on the variables selected, please refer to the SI).

SSFA^107^ was designed to capture the multiscale nature of tooth–food interaction^87,105^ and is often considered the most effective tool for dietary reconstruction^74,76^. Commonly used parameters include Asfc (complexity), Smc, epLsar (anisotropy), HAsfc (heterogeneity), and Tfv^87^. Originally computed using Toothfrax and Sfrax^87,108^ software, these are now implemented in MountainsMap (Digital Surf), which includes more scale-sensitive variables and a corrected NewEpslar but excludes Tfv^108^. Importantly, outputs from different software are not directly comparable despite their similar discriminative power^108^. In this study, SSFA values were generated *ex novo*, avoiding compatibility issues.

ISO 25178-2/ISO 12781 parameters provide standardized indices describing surface texture^97,98^, and have been used in 3DST analyses to reflect food-enamel interactions^74^. They offer an alternative to SSFA for dietary analysis^16,17,69,70,74,109^, though they are not directly comparable^105^. Some studies have combined both types of variables^99,109–111^, yet no systematic comparison exists^68^. In pitheciids, combining both sets facilitated detection of subtle dietary differences^99^.

Finally, in addition to SSFA and ISO variables, we include 8 variables (labeled Other) related to Fourier analysis (*e.g.*, isotropy, directionality, periodicity), and furrow-related features previously used in lithic use-wear^103,111–113^ and dietary inference^67,78,102^.

### Data preprocessing

**Fig. 4:**
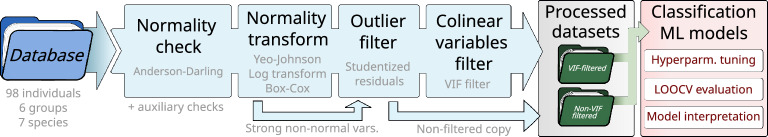
Overview of the complete preprocessing and modeling pipeline. preprocessing steps are blue, with extra arrows indicating (1) the preservation of highly non-normal variables and (2) the non-VIF-filtered copy of the preprocessed dataset. Processed datasets are in green, while the schematic modelling and classification phase is in red. Best viewed in color.

This study aims to develop a standardized data preprocessing pipeline for reliably identifying key variables in primate species classification. To ensure consistent input for classification models, the complete dataset underwent the preprocessing pipeline, schematically represented in Fig. 4, consisting of the following steps:

1. *Checking for normality*. Since some models (*e.g.*, LDA) assume or benefit from normally distributed variables^80^, we assessed normality using visual tools (boxplots, Q-Q plots) and the Anderson-Darling test ($\alpha = 0.05$)^114^, selected for its statistical power and suitability for our sample size.
2. *Transforming for normality*. Variables failing the normality test were transformed to approximate normality. While the $\log$ transform is standard in species classification^16^, we used the Yeo-Johnson transformation^115^ due to its effectiveness with zero and negative values. This generalizes Box-Cox and includes the $\log$ transform as a special case^116^. For a variable *x* and transformation parameter $\lambda$ (estimated via maximum likelihood,^50,115^), it is defined as:
  $$T(x,\lambda )= {\left\{ \begin{array}{ll} \frac{(x+1)^\lambda - 1}{\lambda }, & x\ge 0,\ \lambda \ne 0,\\ \log (x+1), & x\ge 0,\ \lambda =0,\\ -\frac{(-x+1)^{2-\lambda } - 1}{2-\lambda }, & x< 0,\ \lambda \ne 2,\\ -\log (-x+1), & x<0,\ \lambda =2. \end{array}\right. }$$
3. *Detecting and removing outliers*. Following^80^, we flagged and removed outliers (characterized there as points with studentized residuals $\ge 3$) since such extreme observations can disproportionately influence model fitting and reduce classification accuracy. This is particularly important in our leave-one-out measurement framework, where each point serves as a test case once, making outliers especially impactful on classification accuracy. Overall, only a small number of cases were affected, with only six points removed from the dataset.
4. *Dealing with colinear variables*. To address collinearity, which affects models like LDA, we computed the variance inflation factor (VIF,^79^) after standardization, removing variables with VIF $\ge 2$^117^.

These steps form the core of the workflow summarized in Fig. 4, which presents the transformation, filtering, and modeling stages in a unified scheme. The diagram emphasizes the sequential structure of the pipeline and illustrates how preprocessing decisions propagate into model training and interpretation. The preprocessing pipeline is highlighted in blue, with additional connections indicating that we retained variables exhibiting strong non-normal behavior that could not be adequately transformed in the second step. It also reflects our choice to use a version of the processed dataset without applying the VIF filter. These decisions result in two datasets, both of which are subsequently used in the classification tasks for the machine learning models whose results are presented.

The previous steps are critical depending on the algorithm employed. Since not all classification methods are equally sensitive to collinearity, we retain two versions of the dataset: one with and one without the VIF filter. This allows the use of additional information from variables excluded by the collinearity filter when appropriate. Notably, no missing values were reported in this dataset, so no imputation was necessary. If missing data were present, their treatment would need to be evaluated based on their potential impact.

Our pipeline automates most of the preprocessing steps, though certain decisions still require expert judgment. The implementation is tailored to this dataset but can serve as a generalizable framework. For completeness, we store all datasets separately by variable set, as well as a merged version that combines all variables.

### Data analysis

To study the non-normality in the variables, we tested distributions with the Anderson-Darling test: only $12/45$ ISO, $2/20$ SSFA, and $2/8$ Other variables (Fourier and Furrows) appeared normal. Log-transformations had little effect, while the Yeo-Johnson transformation notably improved results, leaving $3$ ISO (Sal, Sdr, FLTq), $8$ SSFA (YMax, SRCth, RegMinS, RegMaxS, RegLS, RLY, RegCo, SMaxComp), and $2$ Other (DomWave, Isotropy2) variables non-normal. Thus, SSFA had the highest proportion of non-normal variables ($40\%$), compared to ISO ($7\%$) and Other ($15\%$). Despite this, non-normal variables were predictive (Fig. 3), so all were retained for optimal performance.

**Fig. 5:**
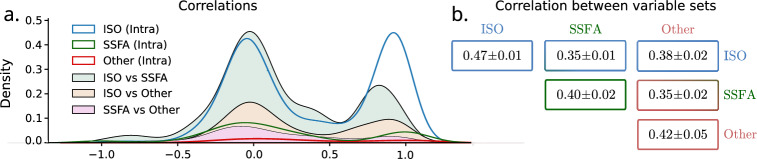
Variable analysis: (*left* - **a**) KDE of correlations for each variable set, both within itself (stroke-only curves) and with other variable sets (color-filled curves). (*right* - **b**) mean absolute correlation across variable sets. Best viewed in color.

We also examined variable correlations both within and across variable sets to assess redundancy within sets and shared information between them. The Kernel Density Estimate (KDE,^50^) plots in Fig. 5 reveal bimodal intra-set distributions, with most variables being either uncorrelated or strongly positively correlated. Cross-set correlations were generally centered near zero, indicating that the sets provide complementary information. Mean absolute correlations further confirmed that intra-set dependencies are stronger than inter-set dependencies. These findings underscore the value of combining variable sets to achieve a richer data representation and improve classification performance (see SI for details).

Finally, we also studied potential outliers and collinearities. The studentized residuals filter removed 6 outliers, and the VIF filter identified collinearities within variable sets. After filtering, $9$ ISO variables (Sku, Str, Std, Shv, Smr1, Sfd, Sci, Smean, and Stdi), $7$ SSFA variables (FractDim, RegMinS, RegCo, CustHAsfc, MadAsfc, Eplsar, New_Eplsar), and $4$ Other variables (MaxDensF, Isotropy1, DomWave, Isotropy2) remained, indicating substantial co-dependence within each set. When applying collinearity filtering to the complete dataset, $13$ variables remained: $7$ from ISO (Sku, Sal, Std, Smr1, Sci, Smean, Stdi), $2$ from SSFA (RegMinS, RegCo), and $4$ from Other (Isotropy1, Amplitude, DomWave, New_Eplsar). This suggests many SSFA variables provide limited additional information. Since model sensitivity to collinearity varies, we retained two dataset versions: one VIF-filtered and one unfiltered, applying all classification models to both. Results are reported for the best-performing configuration.

### Modeling and evaluation

Once the dataset has been preprocessed, we analyze it with the primary goal of correctly classifying primate *groups* (or *species*) based on dental textures as proxies for dietary habits. Since no single classifier can be universally best^118^, our pipeline includes several multi-class classifiers well documented in the literature, *e.g.*,^81^, including *Extreme Gradient Boosting* (*XGBoost*^119^) and *Natural Gradient Boosting* (*NGBoost*^120^). Given our focus on interpretability, we exclude neural network-based models^121^, which typically require larger datasets, extensive hyperparameter tuning, and high computational cost, without offering greater interpretability relevant to our objectives^50^. Specifically, the classifiers included in the pipeline, ordered by decreasing interpretability (and increasing flexibility), were:

- *Linear discriminant analysis* (**LDA**^50^) introduced as the typical baseline in dental data-based classification, as in the originating study^16^.
- *Quadratic discriminant analysis* (**QDA**^50^) extends LDA by using quadratic separating functions and allowing different class covariance matrices. While it introduces more parameters than LDA, the formulation remains similar. Despite being an extension of LDA, QDA has not been widely applied in species classification^103,122^.
- *Naive Bayes* (**NB**), serving as a baseline with minimal analyst intervention, constrained by the parametric form of the distributions selected and the strong structural assumption of conditional independence between variables for each separate class^50^.
- *Multinomial logistic regression* (**MLR**) is a multiclass extension of logistic regression. We use it with regularization terms, specifically *Lasso* and *ElasticNet*, to simplify the model and promote variable selection^50^. The *Lasso* adds an $\ell _1$ penalty, forcing some coefficients to zero and yielding a sparse, interpretable model. The *ElasticNet*, combining $\ell _1$ and $\ell _2$ penalties, is less sparse but better handles correlated predictors. Both methods can also be viewed as maximum-a-posteriori (MAP) estimates, offering a Bayesian perspective on sparsity.
- *Random Forest* (**RF**), another baseline requiring minimal analyst intervention drawing on the idea of decorrelating classification trees^123^. Besides, it requires more hyperparameter tuning than previous approaches.
- *Boosting methods* constitute another general family of methods showing excellent performance in various settings, being also robust to overfitting. Although we tested also *gradient boosting*^124^ and *AdaBoost*^125^, based on the results, we chose to report the metrics just for *XGBoost* (**XGB**^119^) and *NGBoost* (**NGB**^120^), as they provided the best results within this family. However, they require an extensive hyperparameter tuning process.
- *Support vector machines* (**SVMs**^50^) offer a robust and interpretable alternative, relying on support vectors. Originally designed for binary classification, we apply a *one-vs-rest* strategy for multiclass classification. This involves constructing $n_{classes} \times (n_{classes} - 1) / 2$ classifiers, which are then combined for the one-vs-rest approach. We tested *linear*, *RBF*, *polynomial* (degrees 2, 3, and 4), and *sigmoid* SVM kernels.

We used the implementation in &lt;span fontcategory=“NonProportional” name=“Emphasis” class=“NonProportional”&gt;sklearn^126^ for almost all methods, except for *XGBoost* and *NGBoost* for which we employed the original implementations by their authors (XGB code and NGB code).

To better understand variable importance, we focus on MLR models, whose coefficients directly indicate the relevance of each variable to the model’s prediction. Specifically, we analyze the fitted parameters from MLR models for both classification tasks (*groups* and *species*), where the sign of each coefficient shows the direction of association with a class, and its magnitude reflects the strength of that contribution. This allows identification of variables that consistently contribute across tasks or are uniquely important for specific classes, as detailed in Section “Results”.

Classifier performance was assessed using several *metrics* to provide a comprehensive evaluation. Given the manageable dataset size, performance measures were estimated via leave-one-out cross-validation (LOO-CV) on the entire dataset, training each model on all but one data point, evaluating on the held-out point, and averaging the results across all data points. This enables full utilisation of data and provides low-bias estimates of model performance, particularly generalisation error. Although LOO-CV may yield slightly lower performance estimates compared to fixed test sets, it offers a reliable and conservative evaluation by reducing variability and minimising overestimation due to arbitrary train-test splits. The chosen metrics were:

- *Accuracy* (Acc.): Defined as the ratio of correctly classified examples to the total number of instances in the dataset, also used for hyperparameter selection when necessary.
- *Recall* (Rec., *sensitivity*): Represents the fraction of instances correctly classified by the method (true positives divided by sum of true positives and false negatives). Specifically, it is the ratio of correctly classified primates in each class to all individuals in that class.
- F1-*score* (*F*1): A common classification metric, defined as the harmonic mean of precision and recall: $\text {F}1 \,=\, 2 \times \frac{\text {precision} \times \text {recall}}{\text {precision} + \text {recall}}.$ Precision is the proportion of predicted positives that are correctly classified, and recall is the proportion of actual positives correctly identified. The F1-score provides a robust assessment of model performance, being high only when both precision and recall are high.
- *Cohen’s Kappa* (C-$\kappa$): A robust measure of the agreement between the classifier and actual labels beyond chance. It quantifies the proportion of predictions not explained by random guessing, defined as $\kappa = (p_o - p_e)/(1 - p_e)$, where $p_o$ is the observed agreement (accuracy) and $p_e$ is the expected agreement by chance, calculated in the multiclass case as $p_e = N^{-2} \sum _{k=1}^K n_{k} p_{k}$, with *K* categories, *N* observations, $n_k$ the predicted proportion for class *k*, and $p_k$ the actual proportion. The measure ranges from $-1$ to $+1$, with higher values indicating greater agreement, zero indicating random guessing, and negative values worse than random guessing.
- *AUC-ROC* (Area Under the ROC curve) is a common metric for evaluating classification performance. In multiclass classification, it is computed using the *one-vs-rest* approach, where each class is treated as positive in turn, and the rest as negative. The overall AUC-ROC score is the average of these individual values, reflecting the classifier’s ability to discriminate between classes. Scores close to 1 indicate better performance.

For more information on each metric, please see^50^. In all metrics we computed their *macro-average* values by calculating the metric for each class (group or species) and then averaging these across all classes. However, for Cohen’s Kappa and AUC-ROC, we used the *micro-average* values. Micro-averaging aggregates contributions from all classes, considering the total true positives, false positives, and false negatives, which we found to be more stable due to the high variance caused by the small number of instances per class. For accuracies, we report both micro ($\upmu$-*Acc.*) and macro (*M-Acc.*) averages to provide broader context for the pipeline’s results.

To ensure unbiased estimates of model generalization and prevent data leakage, we employed a two-stage nested cross-validation strategy. Hyperparameter optimization was strictly confined to an inner loop utilizing 5-fold cross-validation, with accuracy serving as the primary metric to evaluate the parameter spaces for each method. For models with highly complex hyperparameter spaces (*e.g.*, RF, XGB, NGB), we implemented randomized search cross-validation to efficiently navigate the combinations within a viable computational budget, ensuring fair comparisons across methods. Conversely, models such as LDA, QDA, and Naive Bayes required no tuning, while Multinomial Logistic Regression (MLR) involved evaluating specific regularization paths for the Lasso and ElasticNet penalties. The hyperparameter space for SVMs is detailed above, while in tree-based models optimization focused on critical architectural parameters, *e.g.* the number of estimators, maximum depth, minimum samples to split a node, and bootstrap settings. Once the optimal configurations were identified via this 5-fold tuning phase, the final predictive performance of each model was independently evaluated using Leave-One-Out Cross-Validation (LOOCV), thereby maximizing the training data utilized for our final anthropological assessments. This approach ensures fair evaluation across methods while maintaining accessibility, allowing the pipeline to run without specialized computational resources. It also reflects considerations of computational efficiency and energy consumption, while producing reliable performance estimates and supporting interpretability studies. For full details on the final models’ parameters, please resort to the code repository (https://github.com/simonrsantana/ml_for_primate_dietary_classification/tree/master).

## Supplementary Information

Supplementary Information 1.

Supplementary Information 2.

## Data Availability

Data is provided within the manuscript’s supplementary information files
