# Supplementary material for: Machine learning approaches to dietary classification from dental microtexture in primates
Source: Sci Rep. 2026 Apr 28;16:23378. doi: 10.1038/s41598-026-47350-8 (PMC13408360; doi:10.1038/s41598-026-47350-8)
Supplement: Supplementary file 1 — Supplementary Information 1. [file 41598_2026_47350_MOESM1_ESM.pdf]

Supplementary Information: Machine Learning Approaches to Dietary Classification from Dental Microtexture in Primates

Ferran Estebaranz-Sánchez[1], Kristina Kit[2], Juan José Ibáñez Estevez[3], David R. Insua[2], Simón Rodríguez Santana[4,*], and Laura M. Martínez[5,†]

[1]Archaeology of Social Dynamics, IMF-CSIC, Egipcíaques 15, 08001, Barcelona, Spain
[2]DataLab, Institute of Mathematical Sciences, ICMAT-CSIC, 28049, Madrid, Spain
[3]Cultural Landscape Research Group. Milà i Fontanals Institution. IMF-CSIC, 08001, Barcelona, Spain
[4]ICAI Engineering School, Universidad Pontificia de Comillas - IIT, 28015, Madrid, Spain
[5]Departament de Biologia Evolutiva, Ecologia i CC Ambientals. Facultat de Biologia. Universitat de Barcelona, Institut d'Arqueologia de la Universitat de Barcelona, 08007, Barcelona, Spain
[*]srsantana@comillas.edu
[†]lmartinez@ub.edu

A Supplementary Information

A.1 Scale-Sensitive Fractal Analysis (SSFA)

Anisotropy (exact-proportion length-scale anisotropy of relief, epLsar) represents the degree to which features share a similar orientation (e.g., lots of parallel striations yield more anisotropic surfaces; anisotropy is the mean vector length resulting from quantifying the length and direction of profiles taken at 5° (for a total of 36 measures at each scale).

Complexity(area-scale fractal complexity, Asfc) assesses the change in surface roughness across changing scales of observation, distinguishing taxa consuming brittle foods from taxa consuming softer and/or tougher ones.

The scale of maximum complexity (Smc) measures the fine-scale limits of the Asfc with greater Smc surface values associated with fewer small features.

Heterogeneity (HAsfc(3×3) and HAsfc(9×9)), the degree of texture complexity variation, is measured by calculating the Asfc variation among subdivided samples (a 3 × 3 and 9 × 9 grid, totalling 9 and 81 subsamples, respectively). Thus, surfaces with high heterogeneity have greater disparity in complexity values between subdivided samples and the entire surface.

Textural fill volume (Tfv) measures the volume filled by large (10 µm diameter) and small (2 µm diameter) square cuboids, with high Tfv values indicating potentially deeper and/or larger features (Scott et al 2006, Schubert et al 2010).

Parameter	Unit	Definition
MadAsfc	no unit	Median absolute deviation of Asfc (3x3)
RLY	μm^2	Regression Line Y-intercept
HAsfc81	no unit	Heterogeneity of Asfc (9x9)
MedHAsfc	no unit	Median of Asfc (3x3)
StdDevAsfc	no unit	Standard deviation of Asfc (3x3)
SRC	µm	Smooth-rough crossover
RegMaxS	no unit	Maximum slope of the regression line with selected ranges
New_Eplsar	no unit	Length-scale anisotropy (1.8 µm, 5°)
Smean	no unit	Mean surface slope
SRCth	no unit	Smooth-rough crossover threshold
FDimen	no unit	Fractal Dimension from SSFA regression
RegCO	no unit	Correlation coefficient of the linear regression in log-log plot
Eplsar	no unit	Length-scale anisotropy (Sfrax) (1.8 µm, 5°)
YMax	no unit	Maximum value of the relative-length
RegMinS	no unit	Minimum slope of the regression line with selected ranges
FractDim	no unit	Global Fractal Dimension
RegLS	no unit	Regression Line Slope
FComplex	no unit	Functional Complexity Index
SMaxComp	μm^2	Maximum value of complexity parameters over all scales
Mean HAsfc	no unit	Mean of Asfc (3x3)
Stdi	no unit	Texture direction index
HAsfc9	no unit	Heterogeneity of Asfc (3x3)
CustHAsfc	no unit	Custom heterogeneity of Asfc

A.2 Furrows and Fourier Parameters

Furrows analyze, detect and visualize furrows above or below a threshold height – calculate furrow parameters: max depth, mean depth and mean density.

Fourier analysis decomposes surface texture profiles into their constituent spatial frequencies, allowing the quantification of surface features at different scales. The resulting wavelength parameters describe the characteristic spatial periodicities of surface roughness, reflecting the size and distribution of texture features.

Parameter	Unit	Definition
Isotropy1	°	First direction
Amplitude	μm^2	Maximum peak-to-valley height measured on the surface
DomWave	µm	Dominant wavelength
MaxAmpl	μm^2	Maximum height difference within the surface
Isotropy2	°	Second direction
MaxDepthF	µm	Maximum depth of furrows
MeanDepthF	µm	Mean depth of furrows
MeanDensF	cm/cm^2	Mean density of furrows

A.3 3D Surface Texture (3DST)

Height parameters: quantify the distribution of height values along the z-axis

Parameter	Unit	Definition	ISO
Sq	µm	Root-Mean-Square height of surface	ISO 25178
Sp	µm	Maximum peak height of surface	ISO 25178
Sv	µm	Maximum valley depth of surface	ISO 25178
Sz	µm	Maximum height of surface	ISO 25178
Sa	µm	Average height of surface height	ISO 25178
Ssk	n/a	Skewness of height distribution of surface height	ISO 25178
Sku	n/a	Kurtosis of height distribution of surface	ISO 25178
S10z	µm	Ten-point height	ISO 25178
S5p	µm	Five-point peak height	ISO 25178
S5v	µm	Five-point pit height	ISO 25178
Sxp	µm	Extreme peak height, difference in height between the q and p material ratio (p = 50%, q = 97.5%)	ISO 25178
FLTt	µm	Peak to valley flatness deviation of the surface (Gaussian Filter, 0.025 mm)	ISO 12781
FLTp	µm	Peak to reference flatness deviation (Gaussian Filter, 0.025 mm)	ISO 12781
FLTv	µm	Reference to valley flatness deviation (Gaussian Filter, 0.025 mm)	ISO 12781
FLTq	µm	Root mean square flatness deviation (Gaussian Filter, 0.025 mm)	ISO 12781

Spatial parameters: quantify the direction and spatial periodicity of the surface

Parameter	Unit	Definition	ISO
Str	n/a	Texture aspect ratio (s = 0.2)	ISO 25178
Std	%	Texture direction	ISO 25178

Area

Parameter	Unit	Definition	ISO
Sda	μm^2	Mean dale area	ISO 25178
Sha	μm^2	Mean hill area	ISO 25178

Hybrid parameters: combine the information present on the x-, y- and z-axes of the surface to quantify aspects of the spatial shape of the data.

Parameter	Unit	Definition	ISO
Sdq	n/a	Root mean square gradient of the surface	ISO 25178
Sdr	%	Developed interfacial area ratio	ISO 25178

Measures of volume: calculated from the areal material ratio curve

Parameter	Unit	Definition	ISO
Vm	$\mu m^3/\mu m^2$	Material volume at a given material ratio ($p = 10\%$)	ISO 25178
Vmp	$\mu m^3/\mu m^2$	Material volume of the peaks of the surface	ISO 25178
Vmc	$\mu m^3/\mu m^2$	Material volume of the core of the surface	ISO 25178
Vv	$\mu m^3/\mu m^2$	Void volume at a given material ratio ($p = 10\%$)	ISO 25178
Vvc	$\mu m^3/\mu m^2$	Void volume of the core of the surface	ISO 25178
Vvv	$\mu m^3/\mu m^2$	Void volume of the valleys of the surface	ISO 25178
Sdv	μm^3	Mean dale volume	ISO 25178
Shv	μm^3	Mean hill volume	ISO 25178

Material ratio

Parameter	Unit	Definition	ISO
Sk	µm	Core roughness depth, Height of the core material	ISO 25178
Spk	µm	Mean height of the peaks above the core material	ISO 25178
Svk	µm	Mean depth of the valleys below the core material	ISO 25178
Smr1	%	Surface bearing area ratio (the proportion of the surface which consists of peaks above the core material)	ISO 25178
Smr2	%	Surface bearing area ratio (the proportion of the surface which would carry the load)	ISO 25178

Peak sharpness

Parameter	Unit	Definition	ISO
Spc	1/µm	Arithmetic mean peak curvature	ISO 25178

Plateau size

Parameter	Unit	Definition	ISO
Smc	µm	Inverse areal material ratio ($p = 10\%$)	ISO 25178

A.4 Variable analysis

Figure 1 presents the full heatmap of correlations between the variables. To enhance interpretability, we categorize them into three sets in colors: ISO (blue), SSFA (green), and Other (red). On the other hand, Figure **??**(a - right) illustrates the mean absolute correlation values and standard error for each variable set. As noted in the main text, intra-set correlations are substantially stronger than cross-correlations between sets, indicating that combining variables from different sets may provide complementary information beyond what each set offers individually. Notably, correlations among ISO and Other variables tend to be stronger than those within the SSFA set.

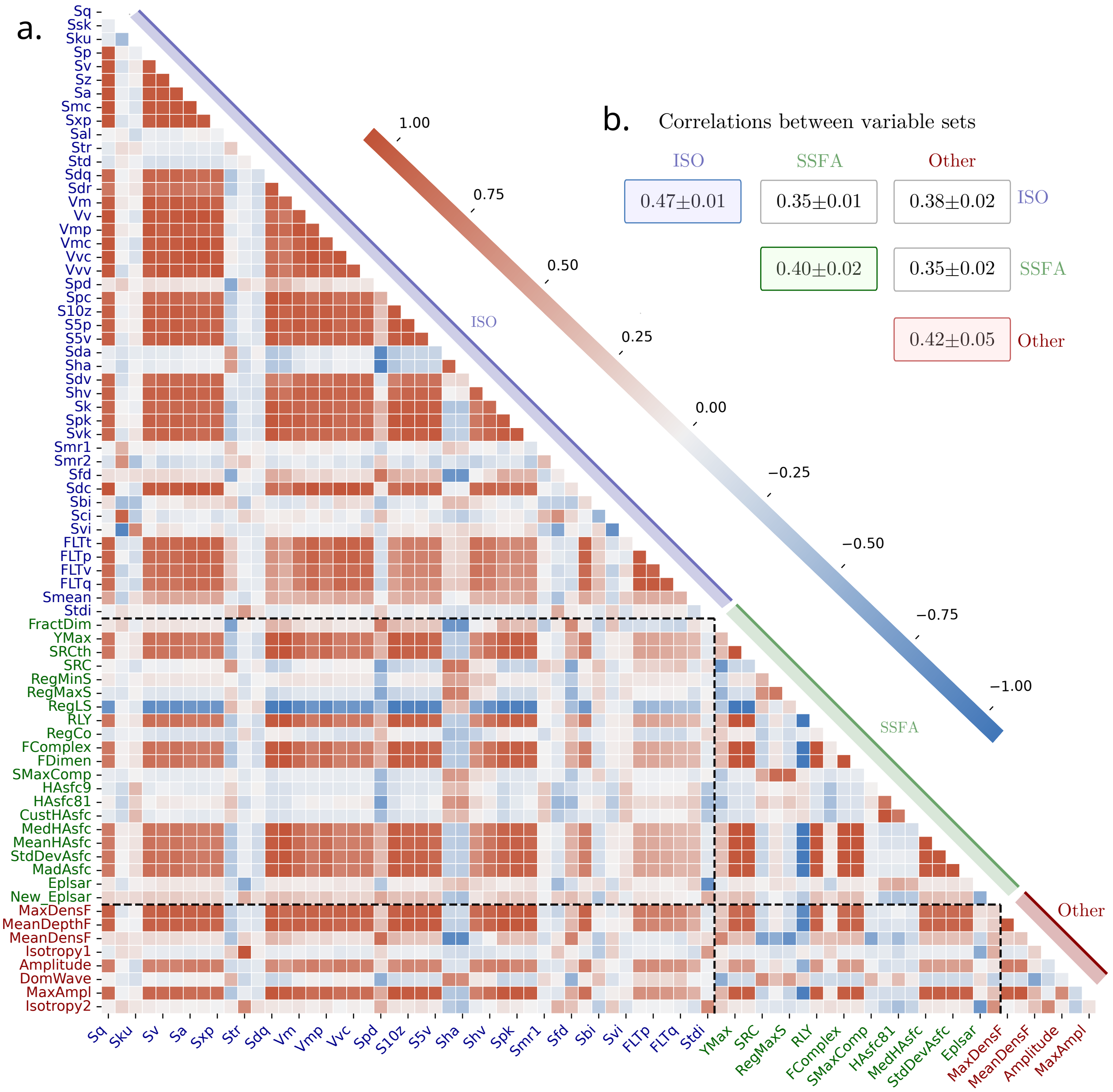

Figure 1. a.: Correlations for all variables. `ISO` variables highlighted in blue. `SSFA` variables in green. `Other` variables in red. **b.**: Top-right values are means and standard errors for the absolute values of correlations among variable sets.

On the other hand, Figure 2 displays the results of the reduced dimensionality analysis using LDA to select the two main directions of the plot, as done in the main body of the article. The left panel depicts the first two LDA components for *groups* showing the same image as the correlations plot in the main text, where each of the six classes forms a relatively distinct cluster, illustrating how these broader taxonomic groups (e.g., *Cercocebus t. atys*, *Chlorocebus*, *Colobus*, etc.) can be separated using these two linear discriminants. In contrast, the right panel shows the first two LDA components for *species*, where the total number of classes increases to seven, causing some classes (especially those that split from the same group) to appear closer together or show greater overlap. Notably, while most species still occupy distinct regions of the plot, the additional class introduces a finer level of detail that can lead to slightly more overlap and less pronounced boundaries in some areas. In this case, the main difference between this plot and the one relative to groups is that *Chlorocebus* samples seem to be clearly distinguished from the rest. Note here that the loadings for the LD components change for each case (groups and species), so that the directions are not directly comparable.

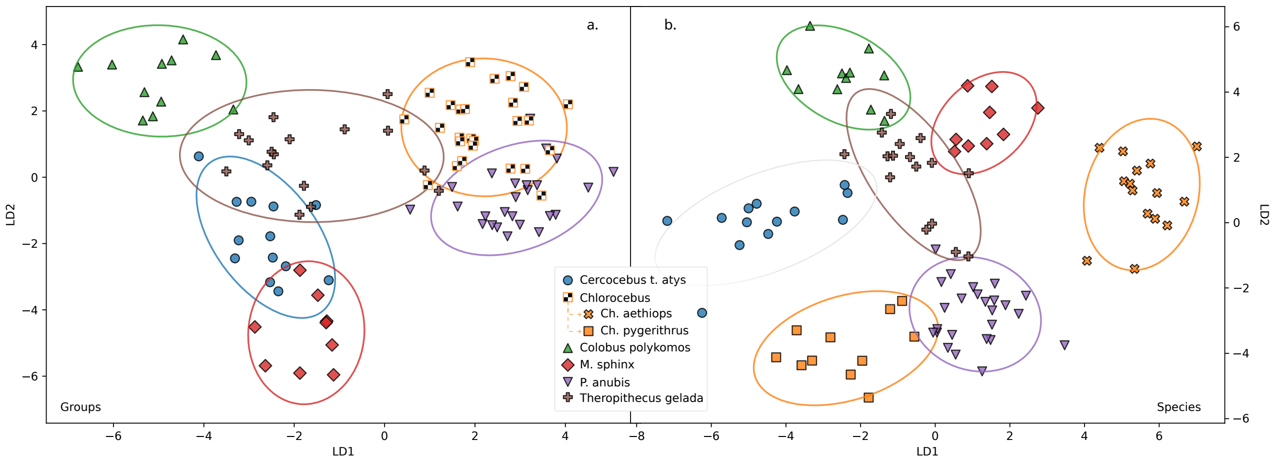

Figure 2. First two LDA components for groups (*left*) and species (*right*). Ellipses indicate the 2σ confidence regions for each label. Note that the LD component loadings differ between both cases, so directions are not directly comparable across group and species plots.

A.5 Extra results for classification

A.6 Classification results for species data

Table 1 presents the classification results for species. Overall, the methods exhibit slightly lower performance levels compared to group classification, which is expected given the increased complexity of distinguishing among seven species instead of six groups. As before, only the best results obtained for each method with each variable set are reported.

In this case, VIF-filtered variables are used selectively in the following scenarios:

- For the `ISO` and `Other` variable sets, filtered variables are applied exclusively with NGB.
- For the `SSFA` variable set, no results are reported using the filtered variables.
- For the `All` variable set, results with filtered variables are reported for both Elastic Net MLR and NGB.

Table 1. Classification results for **Groups**. Each model tuned for optimal performance per variable set.

	Metrics	LDA	LDA$_C$	QDA	MLR	Lasso	EN	NB	SVM	RF	XGB	NGB
	μ-Acc.	0.22	0.26	0.18	0.31	0.30	**0.32**	0.31	**0.35**	0.27	0.24	0.27
	M-Acc	0.20 ± 0.04	0.21 ± 0.07	0.15 ± 0.04	0.28 ± 0.05	0.27 ± 0.06	**0.29 ± 0.06**	**0.30 ± 0.09**	0.28 ± 0.10	0.22 ± 0.07	0.20 ± 0.06	0.23 ± 0.07
I	Prec.	0.20 ± 0.04	0.22 ± 0.06	0.17 ± 0.05	**0.30 ± 0.05**	**0.30 ± 0.07**	**0.30 ± 0.05**	0.24 ± 0.06	0.28 ± 0.10	0.19 ± 0.05	0.21 ± 0.05	0.26 ± 0.08
S	Rec.	0.20 ± 0.04	0.21 ± 0.07	0.15 ± 0.04	0.28 ± 0.05	0.27 ± 0.06	**0.29 ± 0.06**	**0.30 ± 0.09**	0.28 ± 0.10	0.22 ± 0.07	0.20 ± 0.06	0.23 ± 0.07
O	*F*1	0.20 ± 0.04	0.20 ± 0.06	0.15 ± 0.04	**0.29 ± 0.05**	0.28 ± 0.05	**0.29 ± 0.05**	0.25 ± 0.06	0.26 ± 0.08	0.20 ± 0.06	0.19 ± 0.05	0.22 ± 0.05
	C-κ	0.07	0.09	0.02	0.17	0.15	**0.18**	**0.18**	**0.19**	0.11	0.08	0.10
	AUC-ROC	0.58	0.63	0.51	0.67	**0.68**	0.67	0.64	**0.68**	0.67	0.64	0.55
	μ-Acc.	0.12	0.26	0.26	0.25	0.27	0.26	0.19	**0.30**	**0.28**	0.25	0.23
S	*M*-Acc	0.10 ± 0.05	0.20 ± 0.08	0.14 ± 0.14	0.21 ± 0.06	**0.22 ± 0.06**	**0.22 ± 0.05**	0.17 ± 0.05	0.19 ± 0.13	**0.23 ± 0.07**	0.19 ± 0.08	0.18 ± 0.05
S	Prec.	0.09 ± 0.04	0.19 ± 0.05	0.04 ± 0.04	0.19 ± 0.05	0.20 ± 0.05	**0.22 ± 0.05**	0.16 ± 0.05	0.12 ± 0.06	**0.23 ± 0.05**	0.19 ± 0.06	**0.22 ± 0.05**
F	Rec.	0.10 ± 0.05	0.20 ± 0.08	0.14 ± 0.14	0.21 ± 0.06	**0.22 ± 0.06**	**0.22 ± 0.05**	0.17 ± 0.05	0.19 ± 0.13	**0.23 ± 0.07**	0.19 ± 0.08	0.18 ± 0.05
A	*F*1	0.09 ± 0.04	0.19 ± 0.06	0.06 ± 0.06	0.20 ± 0.05	0.21 ± 0.06	**0.22 ± 0.05**	0.16 ± 0.04	0.13 ± 0.08	**0.23 ± 0.05**	0.18 ± 0.06	0.19 ± 0.04
	C-κ	−0.04	0.09	0.00	0.09	**0.11**	**0.11**	0.04	0.09	**0.12**	0.08	0.05
	AUC-ROC	0.54	0.63	0.50	**0.65**	**0.65**	**0.65**	0.59	0.58	**0.67**	0.61	0.56
	μ-Acc.	**0.30**	0.22	0.18	0.26	0.28	0.26	0.18	0.26	0.25	0.19	**0.29**
E	*M*-Acc	**0.24 ± 0.08**	0.17 ± 0.07	0.14 ± 0.06	0.21 ± 0.07	**0.24 ± 0.06**	0.21 ± 0.07	0.14 ± 0.05	0.16 ± 0.12	0.21 ± 0.07	0.12 ± 0.08	**0.24 ± 0.07**
x	Prec.	0.23 ± 0.05	0.17 ± 0.05	0.14 ± 0.05	0.20 ± 0.05	**0.25 ± 0.03**	0.20 ± 0.06	0.14 ± 0.06	0.08 ± 0.05	0.24 ± 0.06	0.07 ± 0.04	**0.25 ± 0.05**
t	Rec.	**0.24 ± 0.08**	0.17 ± 0.07	0.14 ± 0.06	0.21 ± 0.07	**0.24 ± 0.06**	0.21 ± 0.07	0.14 ± 0.05	0.16 ± 0.12	0.21 ± 0.07	0.12 ± 0.08	**0.24 ± 0.07**
r	*F*1	**0.23 ± 0.06**	0.16 ± 0.06	0.13 ± 0.04	0.21 ± 0.06	**0.24 ± 0.05**	0.21 ± 0.06	0.14 ± 0.05	0.10 ± 0.07	0.21 ± 0.05	0.09 ± 0.05	**0.23 ± 0.06**
a	C-κ	**0.15**	0.06	−0.02	0.11	0.13	0.11	0.03	0.05	0.09	−0.02	**0.14**
	AUC-ROC	**0.66**	**0.65**	0.51	**0.65**	**0.65**	**0.65**	0.61	0.49	0.61	0.48	**0.65**
	μ-Acc.	0.16	0.27	0.12	**0.31**	**0.31**	0.30	0.29	0.27	0.29	0.29	0.24
	M-Acc	0.14 ± 0.03	0.22 ± 0.05	0.12 ± 0.03	0.25 ± 0.07	0.25 ± 0.08	**0.26 ± 0.07**	**0.27 ± 0.08**	0.15 ± 0.14	0.22 ± 0.08	0.23 ± 0.07	0.19 ± 0.06
A	Prec.	0.15 ± 0.05	0.22 ± 0.04	0.12 ± 0.03	**0.25 ± 0.06**	0.24 ± 0.07	**0.25 ± 0.05**	0.22 ± 0.07	0.11 ± 0.07	0.20 ± 0.06	0.22 ± 0.05	0.21 ± 0.06
l	Rec.	0.14 ± 0.03	0.22 ± 0.05	0.12 ± 0.03	0.25 ± 0.07	0.25 ± 0.08	**0.26 ± 0.07**	**0.27 ± 0.08**	0.15 ± 0.14	0.22 ± 0.08	0.23 ± 0.07	0.19 ± 0.06
l	*F*1	0.14 ± 0.04	0.22 ± 0.05	0.12 ± 0.02	**0.25 ± 0.06**	0.24 ± 0.07	**0.25 ± 0.06**	0.23 ± 0.07	0.08 ± 0.06	0.20 ± 0.07	0.22 ± 0.06	0.19 ± 0.05
	C-κ	0.01	0.11	−0.04	**0.16**	**0.16**	0.15	**0.16**	0.00	0.12	0.14	0.07
	AUC-ROC	0.50	0.66	0.49	0.67	**0.68**	0.65	0.63	0.28	**0.68**	0.65	0.50

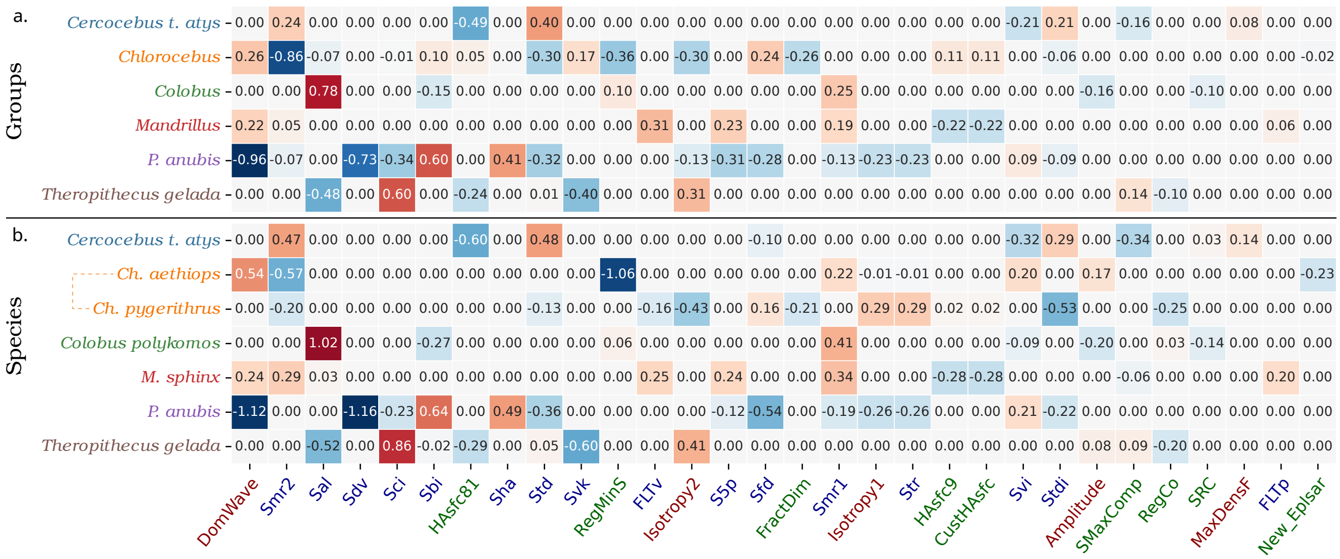

Figure 3. Comparison of raw Lasso MLR coefficients for groups (*top*) and species (*bottom*) classification. Different species and groups are color coded, as well as variable sets: `ISO` in blue, `SSFA` in green and `Other` in red. Best viewed in color.

Taking Table 1 into account, we observe a pattern similar to the results for groups in Table **??** for some variable sets, though with some unexpected behavior in certain cases. In particular, the `ISO` variable set exhibits a performance trend akin to that seen in group classification, where NB and MLR-based models consistently achieve the highest metrics overall. This aligns with the nature of `ISO` variables: they are low-dimensional, relatively less noisy, and often close to conditionally independent, which favors NB, while the moderate collinearity and multiclass structure favor MLR-based models with regularization. Here, LDA and LDA_C also lag in performance, though the gap appears smaller than in the group classification task. This is likely due to the generally lower performance for all models in species classification, which reduces the overall spread in accuracy across methods.

On the other hand, for the `SSFA` variable set, we observe a notable improvement in the performance of the Random Forest (RF) algorithm, which emerges as the best-performing method, closely followed by MLR-based models. This suggests that RF benefits from the higher dimensionality and nonlinear interactions present in SSFA variables, capturing signal that linear methods may not leverage as effectively. At the same time, RF tends to be more robust than NB or LDA to noisy predictors, which may explain its relative advantage in this setting. This underscores the sensitivity of some models to noise and limited sample size, further reinforcing MLR as the most reliable overall choice, given its consistently strong performance across variable sets and tasks. A similar trend appears with the `Other` variable set, where LDA again seems to achieve the best overall performance (mirroring the group classification results) likely because the reduced dimensionality of this set aligns with LDA's assumptions, followed by MLR (this time with Lasso regularization), which remains competitive due to its ability to shrink uninformative coefficients.

Finally, the results obtained using all variables together (`All` dataset) further support the preference for MLR models, as they achieve the best results overall, only slightly outperformed by NB in specific cases. Notably, however, the performance of the `All` variable set remains slightly lower than that achieved with the `ISO` variables alone. This reinforces a key observation discussed earlier: while the `SSFA` and `Other` variable sets may introduce additional information absent in `ISO`, the increased dimensionality and collinearity generally outweigh their benefits in small-sample settings. In this instance, models consistently perform better when using only the `ISO` variables rather than combining all variable sets, regardless of collinearity filtering, in terms of classification accuracy.

Many key variables retain their importance across both classification tasks, reflecting the similarity between them, species classification being a finer-grained version of group classification, where one group splits into two species. However, some differences emerge, such as the stronger role of `RegMaxS` for *Ch. aethiops* compared to its minimal influence for *Ch. pygerythrus*. As noted in the main text, the model selects 32 variables, largely overlapping with those used for group classification, except for `FLTv` and `Spk`, which are replaced by `S5v` and `Sz`. Overall, variables with large absolute coefficients show similar behavior in both tasks, with the main differences attributable to the subdivision of the *Chlorocebus* group.

A.6.1 Permutation importance

To provide a fuller perspective on the variable choices made by different models, Figure 4 presents the permutation importance (PI) analysis for the accuracy of the random forest model trained on the Groups classification task (the Species task yields

qualitatively similar patterns). As in the main text, variables are color-coded by group (`ISO` in blue, `SSFA` in green, and `Other` in red).

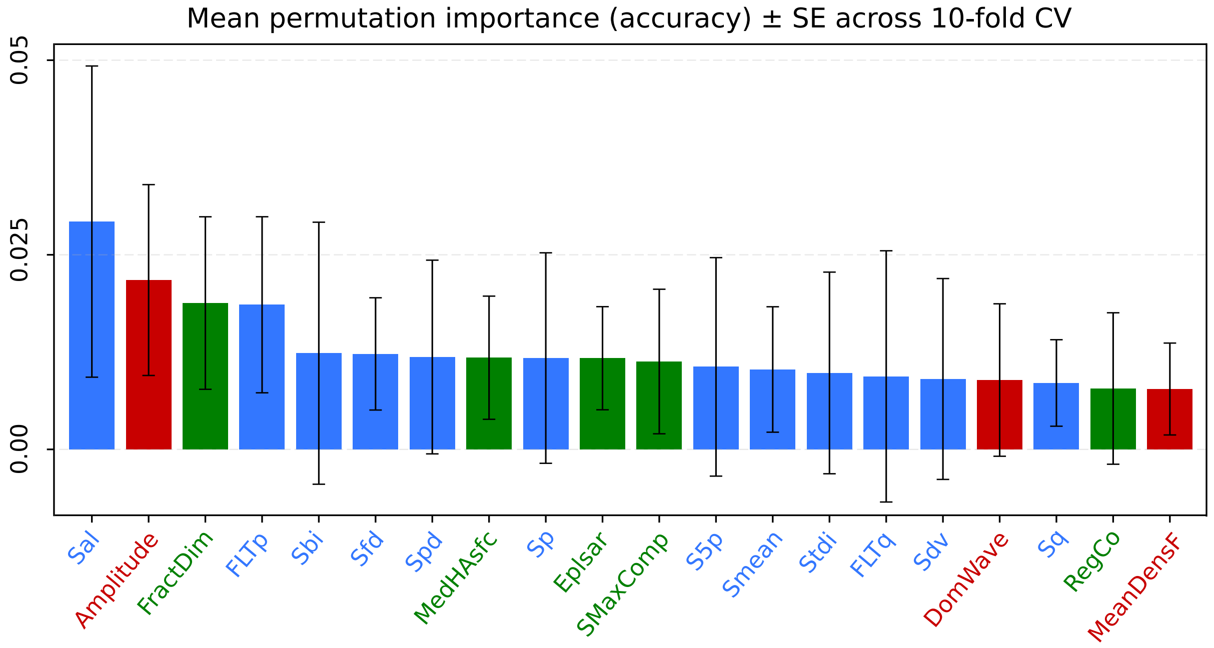

Figure 4. Permutation importance (PI) analysis for the random forest model applied to the accuracy for the group classification task. PI values represent the mean decrease in classification accuracy when each variable is permuted. Estimates are obtained via 10-fold cross-validation with 50 permutations per fold. Error bars indicate the standard error across folds. `ISO` variables are shown in blue, `SSFA` in green, and `Other` in red. Best viewed in color.

Among the top 20 ranked variables, 12 belong to the `ISO` group, 5 to the `SSFA` group, and 3 to `Other`, suggesting that, for the random forest model, `ISO` features collectively contribute more to predictive performance. Notably, some variables that appear here among the more influential ones (e.g., `Amplitude`, `FLTp`, `Spd`) are either absent from or play only a minor role in the MLR-L1 model. This discrepancy is expected, since permutation-based rankings reflect how a non-linear ensemble distributes its predictive capacity across correlated features, whereas L1-regularized linear models select a smaller and often sparser subset.

It is important to emphasize, however, that the magnitude of PI values is generally small (typically on the order of only 0.01–0.03 decrease in accuracy), indicating that no single variable exerts a strong influence on model performance. Moreover, several error bars are comparable to their corresponding means, revealing considerable variability across folds. This instability suggests that feature importances are not strongly reliable at the individual-variable level and may fluctuate substantially with different data splits. Larger datasets or more detailed analysis for these ensemble models could help reduce this uncertainty. However, that falls outside the scope of this paper and would be explored in future contributions.

For these reasons, and because ensemble models tend to be less interpretable, we ultimately prioritize the more transparent and stable coefficient-based interpretation offered by the MLR-L1 model (see Fig. **??**).

A.6.2 Confusion matrix

For the selected MLR–Lasso setting, we construct the corresponding confusion matrix obtained via LOOCV and compare it with the results provided by LDA, which represents the standard approach in previous studies [**?**].

The resulting confusion matrices are shown in Figure 5 and highlight a clear performance difference between the two classifiers. Although LDA has been the standard method in earlier analyses, and despite its performance being enhanced by our preprocessing pipeline, it still exhibits substantial confusion across several species, particularly for *Cercocebus t. atys*, *Colobus*, and *Theropithecus gelada*. In contrast, the MLR model achieves better overall classification results, with higher correct counts for key species such as *P. anubis* and *Chlorocebus*, and fewer diffuse misclassifications overall (notwithstanding the concentration of errors for *Chlorocebus* cases misclassified as *P. anubis*). Although confusion persists between *P. anubis* and *Chlorocebus*, these taxa cohabit the same ecosystems and are opportunistic omnivores occupying distinct ecological niches. However, it is plausible that both taxa ingest similar abrasive particles through their respective food items, generating overlapping buccal microtexture signatures and thereby contributing to their mutual misclassification. Given that LDA represents

a strengthened baseline, the improved structure and concentration of correct predictions in the MLR confusion matrix indicate that MLR captures species-specific patterns more effectively, constituting a meaningful improvement over the traditional approach. When accounting for the number of datapoints per species, the overall performance implied by the MLR confusion matrix is consistent with an accuracy close to 30%, in line with the values reported in Table **??**.

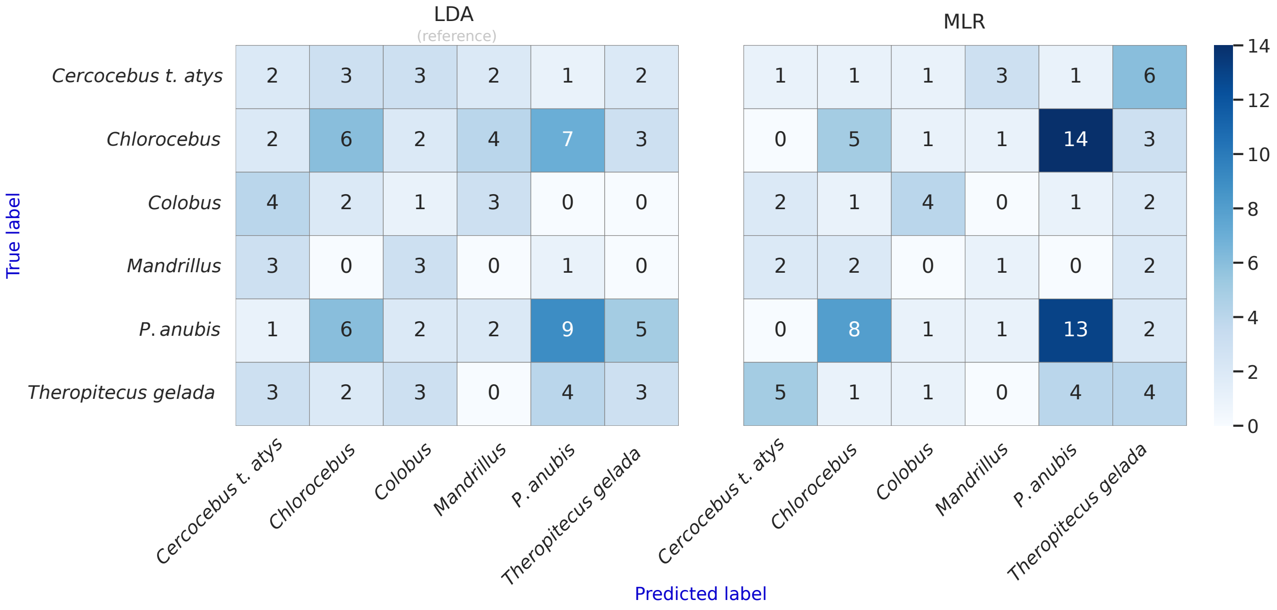

Figure 5. Comparison of the confusion matrices between the LDA model and MLR for group classification using the preprocessed dataset and the setup described in the main text. True labels are included in the left axis, while predicted labels are in the bottom axis.
