# Supplementary figures and images for: Machine learning approaches to dietary classification from dental microtexture in primates
Source: Sci Rep. 2026 Apr 28;16:23378. doi: 10.1038/s41598-026-47350-8 (PMC13408360; doi:10.1038/s41598-026-47350-8)

t-SNE on Reduced Components

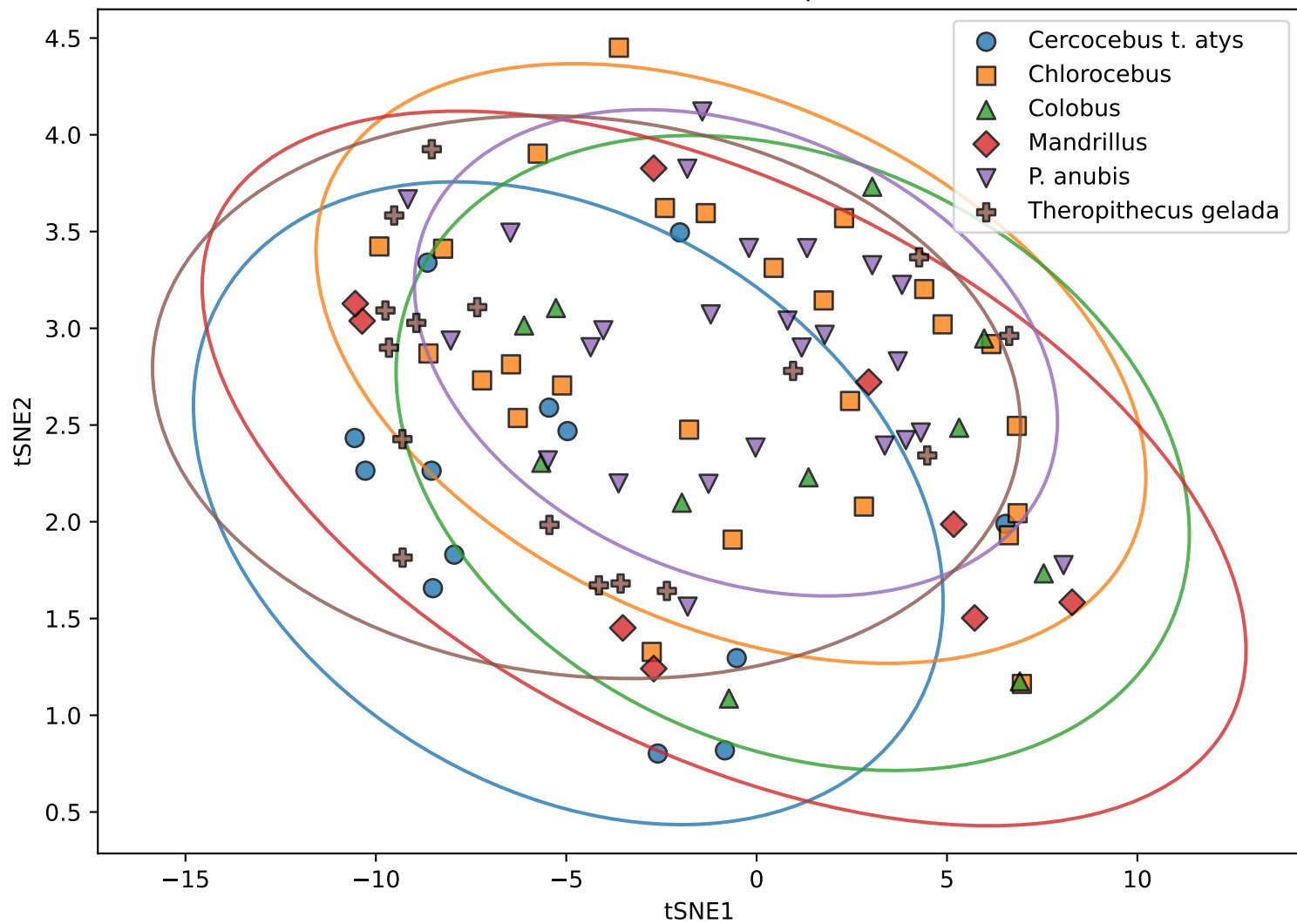

Supplement: Supplementary file 2 — Supplementary Information 2. [file 41598_2026_47350_MOESM2_ESM.zip › code_and_data_new/figures/reduced_dim_pdf/tsne_result_reduced_GENNUS GROUP_pca.pdf]

PCA Result

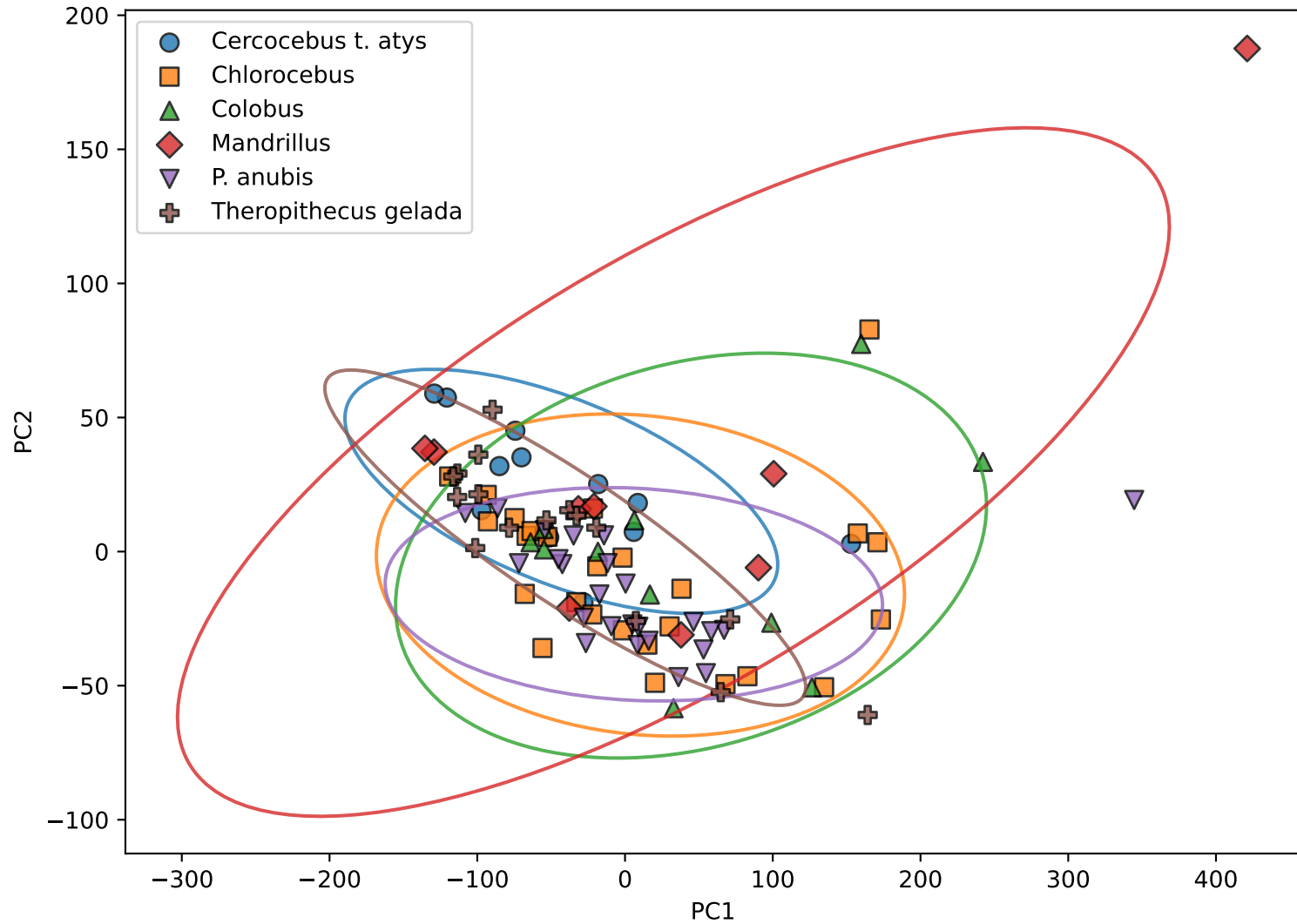

Supplement: Supplementary file 2 — Supplementary Information 2. [file 41598_2026_47350_MOESM2_ESM.zip › code_and_data_new/figures/reduced_dim_pdf/pca_result_GENNUS GROUP_pca.pdf]

UMAP on Reduced Components

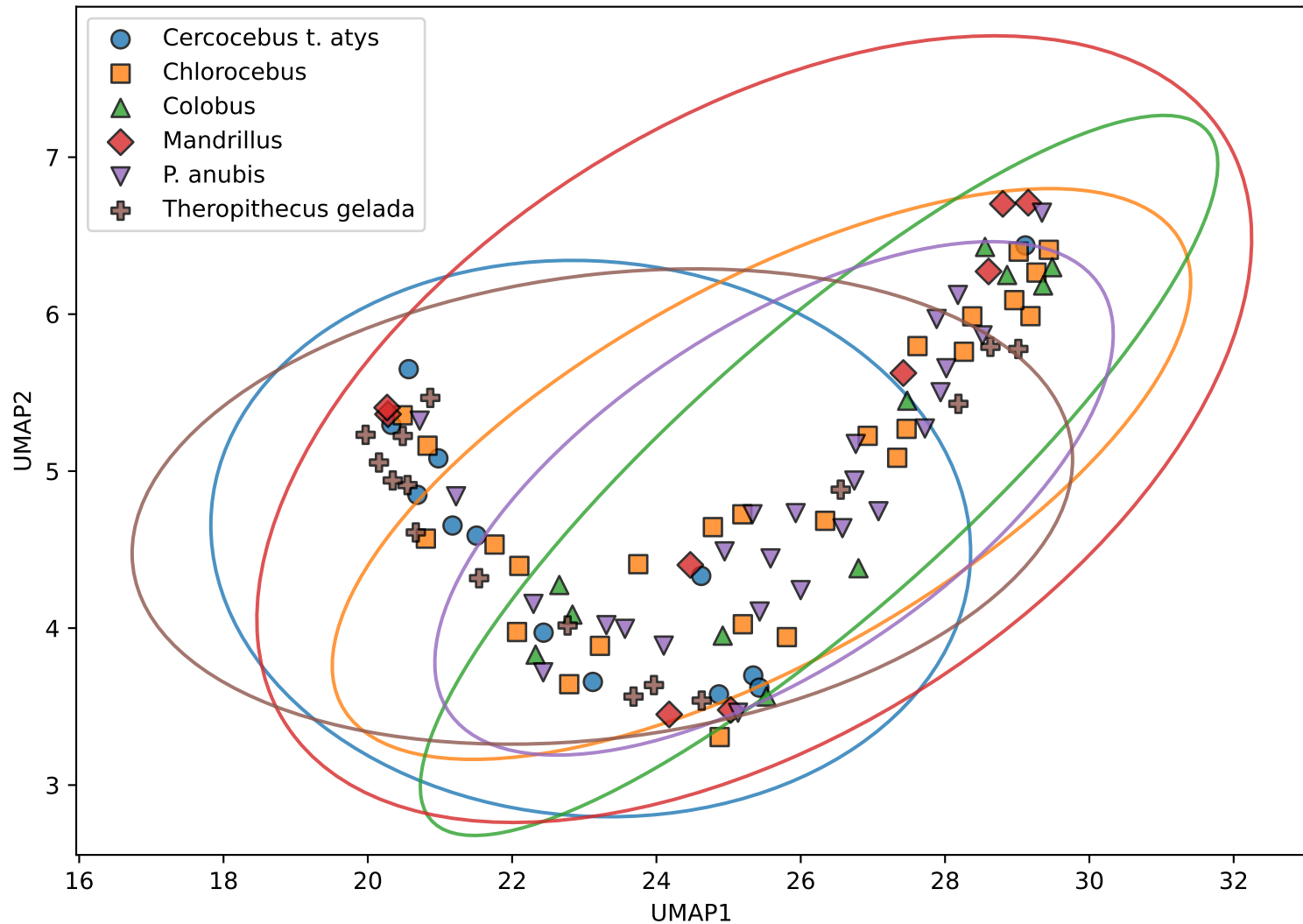

Supplement: Supplementary file 2 — Supplementary Information 2. [file 41598_2026_47350_MOESM2_ESM.zip › code_and_data_new/figures/reduced_dim_pdf/umap_result_reduced_GENNUS GROUP_pca.pdf]

LDA Result

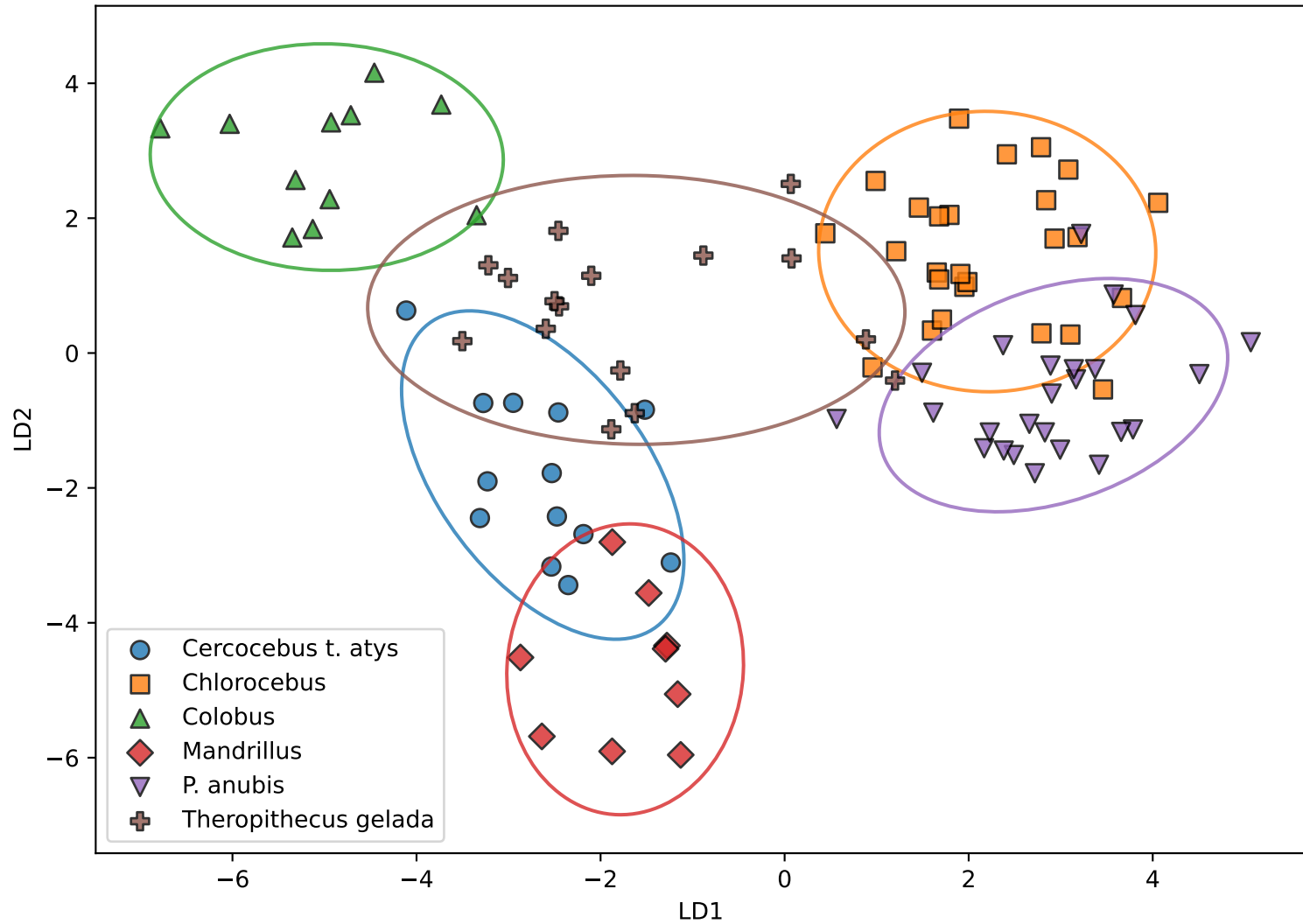

Supplement: Supplementary file 2 — Supplementary Information 2. [file 41598_2026_47350_MOESM2_ESM.zip › code_and_data_new/figures/reduced_dim_pdf/lda_result_GENNUS GROUP_lda.pdf]

UMAP on Reduced Components

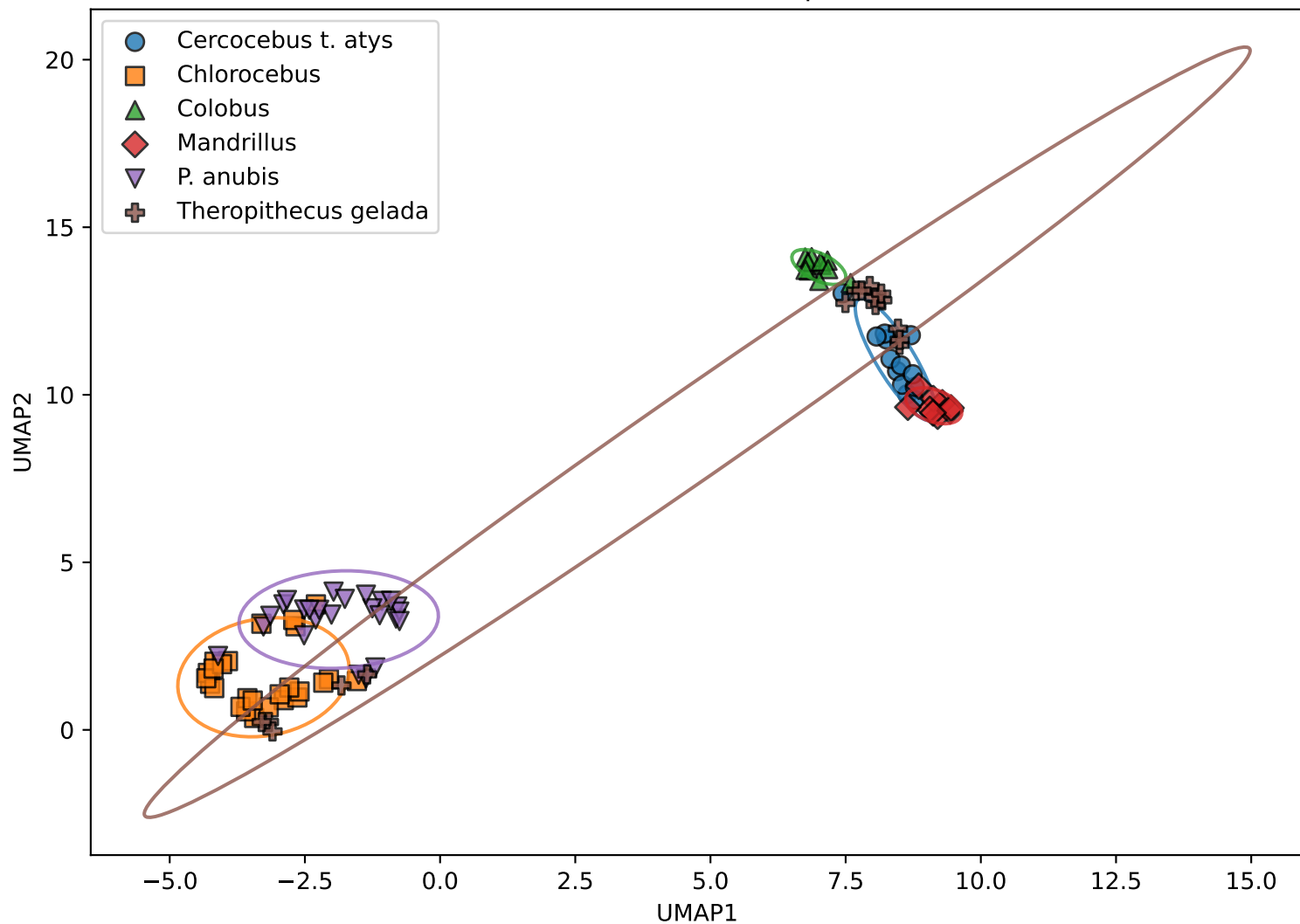

Supplement: Supplementary file 2 — Supplementary Information 2. [file 41598_2026_47350_MOESM2_ESM.zip › code_and_data_new/figures/reduced_dim_pdf/umap_result_reduced_GENNUS GROUP_lda.pdf]

t-SNE on Reduced Components

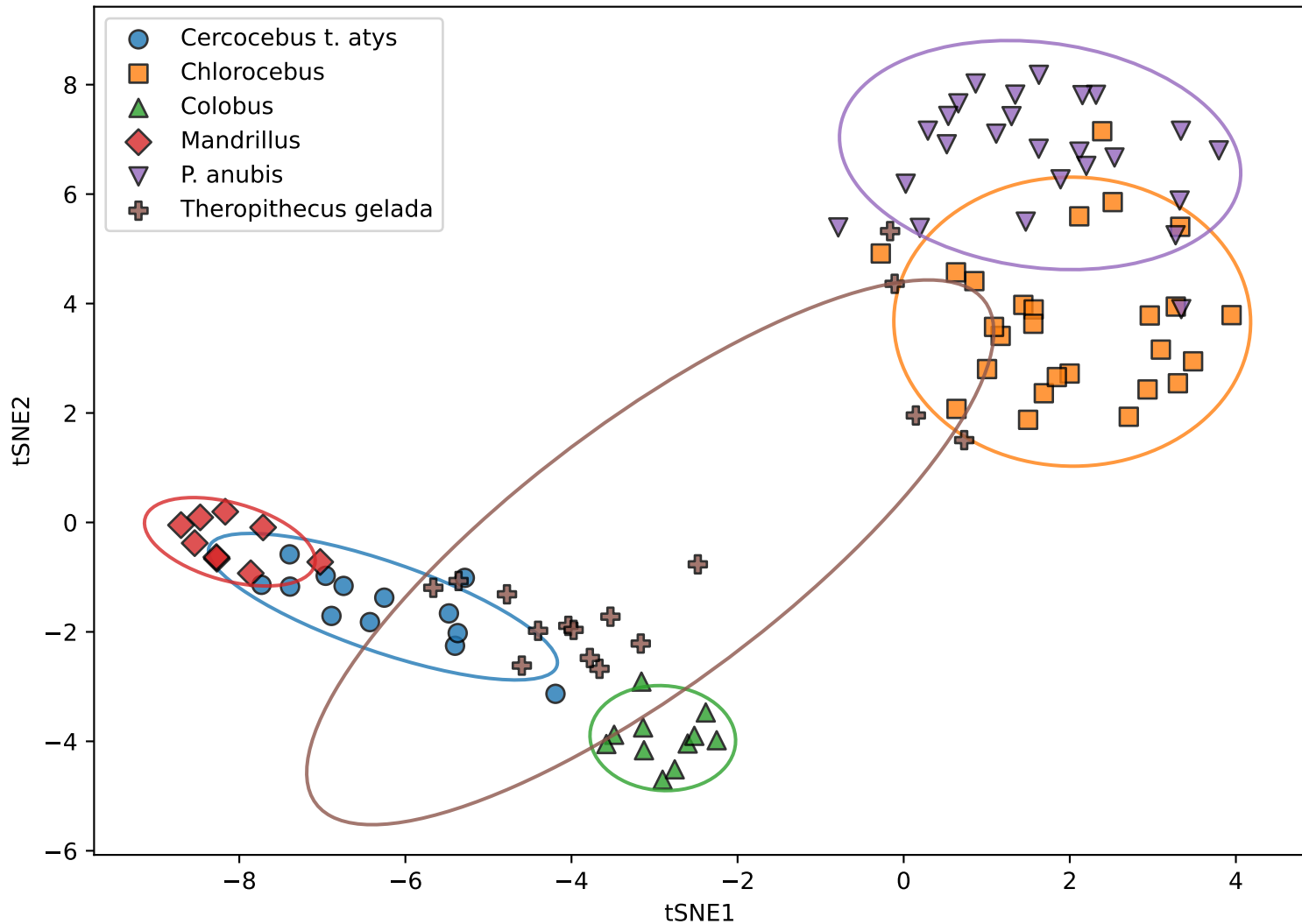

Supplement: Supplementary file 2 — Supplementary Information 2. [file 41598_2026_47350_MOESM2_ESM.zip › code_and_data_new/figures/reduced_dim_pdf/tsne_result_reduced_GENNUS GROUP_lda.pdf]

PCA Result

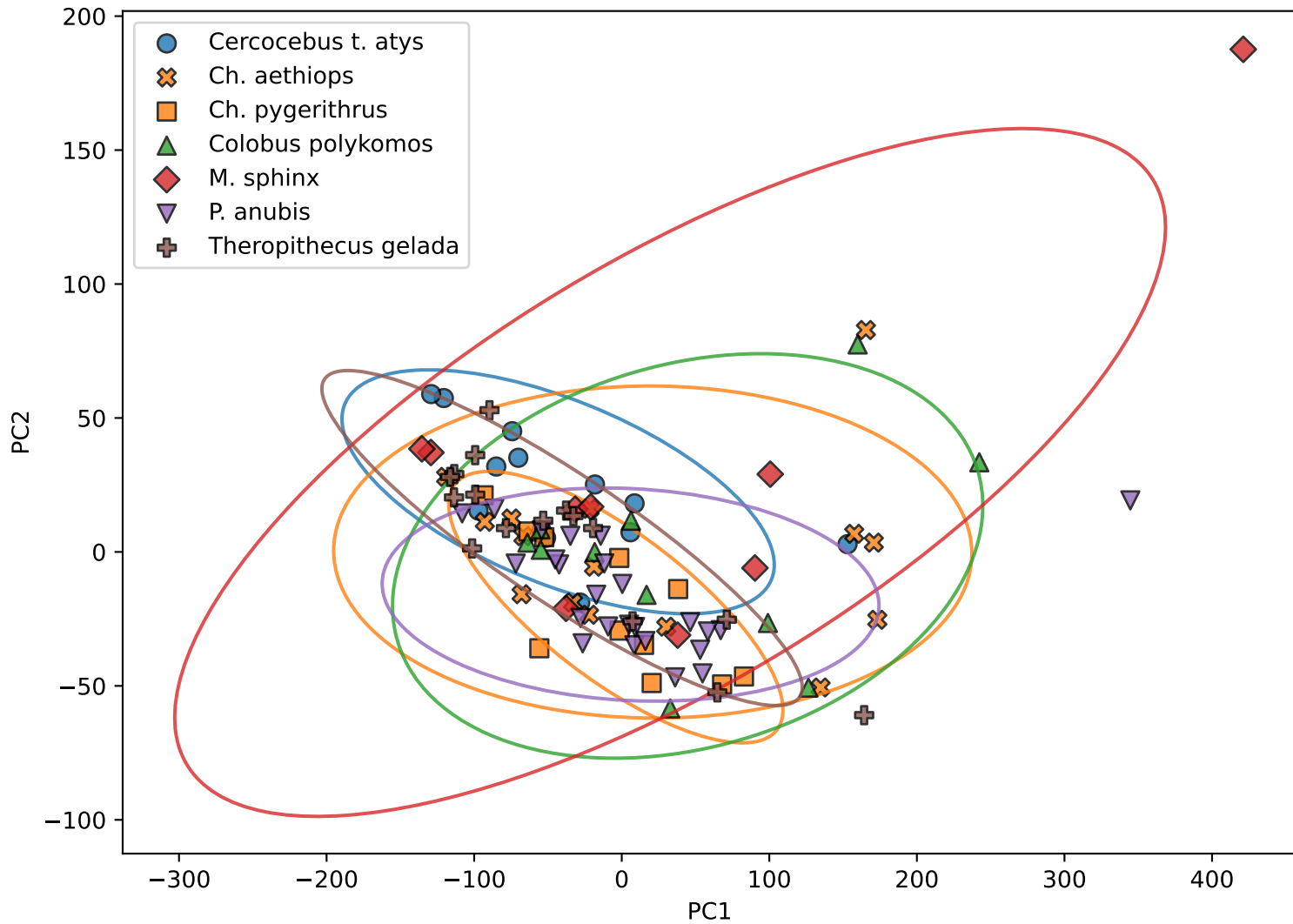

Supplement: Supplementary file 2 — Supplementary Information 2. [file 41598_2026_47350_MOESM2_ESM.zip › code_and_data_new/figures/reduced_dim_pdf/pca_result_SPECIES _pca.pdf]

UMAP on Reduced Components

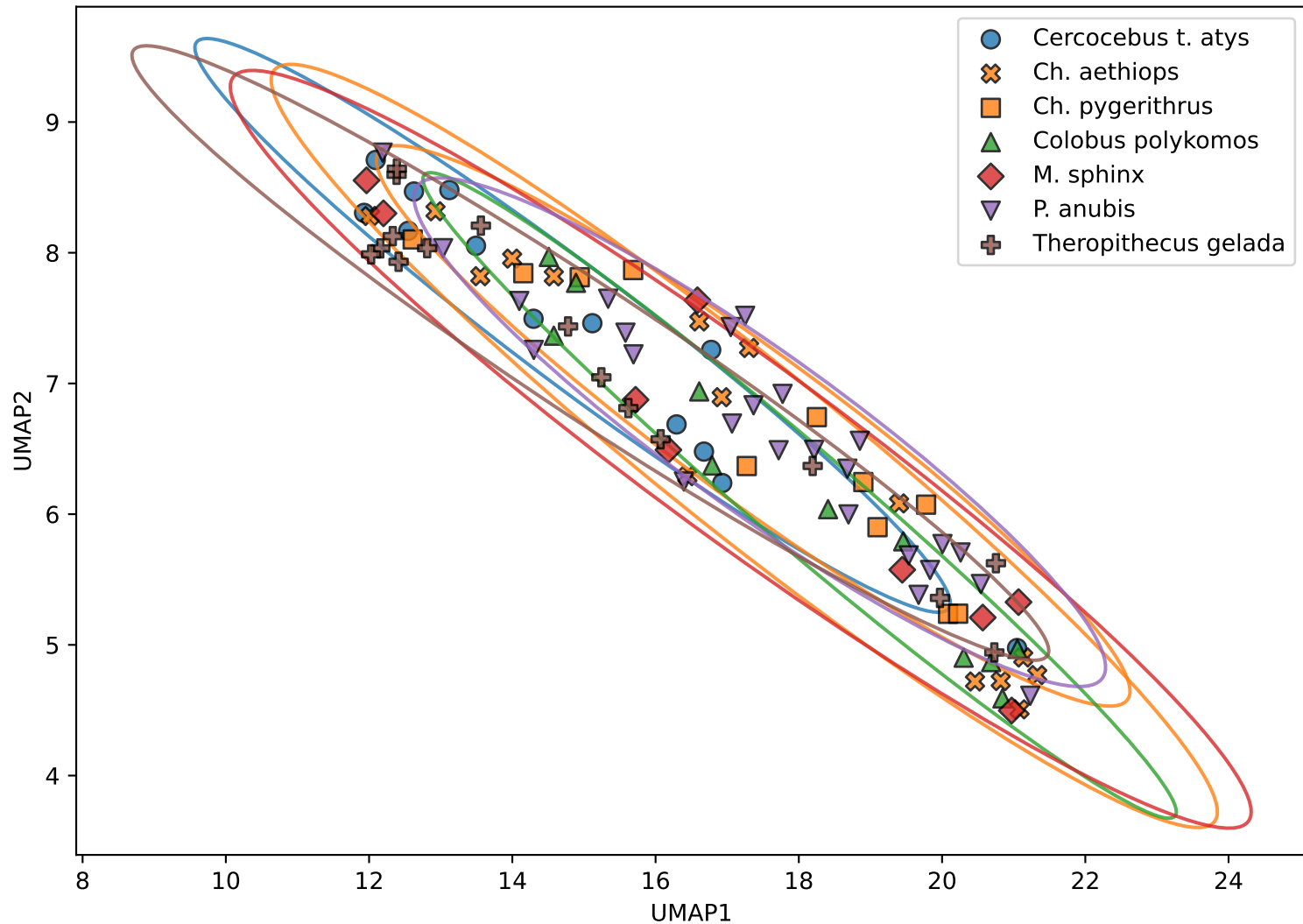

Supplement: Supplementary file 2 — Supplementary Information 2. [file 41598_2026_47350_MOESM2_ESM.zip › code_and_data_new/figures/reduced_dim_pdf/umap_result_reduced_SPECIES _pca.pdf]

t-SNE on Reduced Components

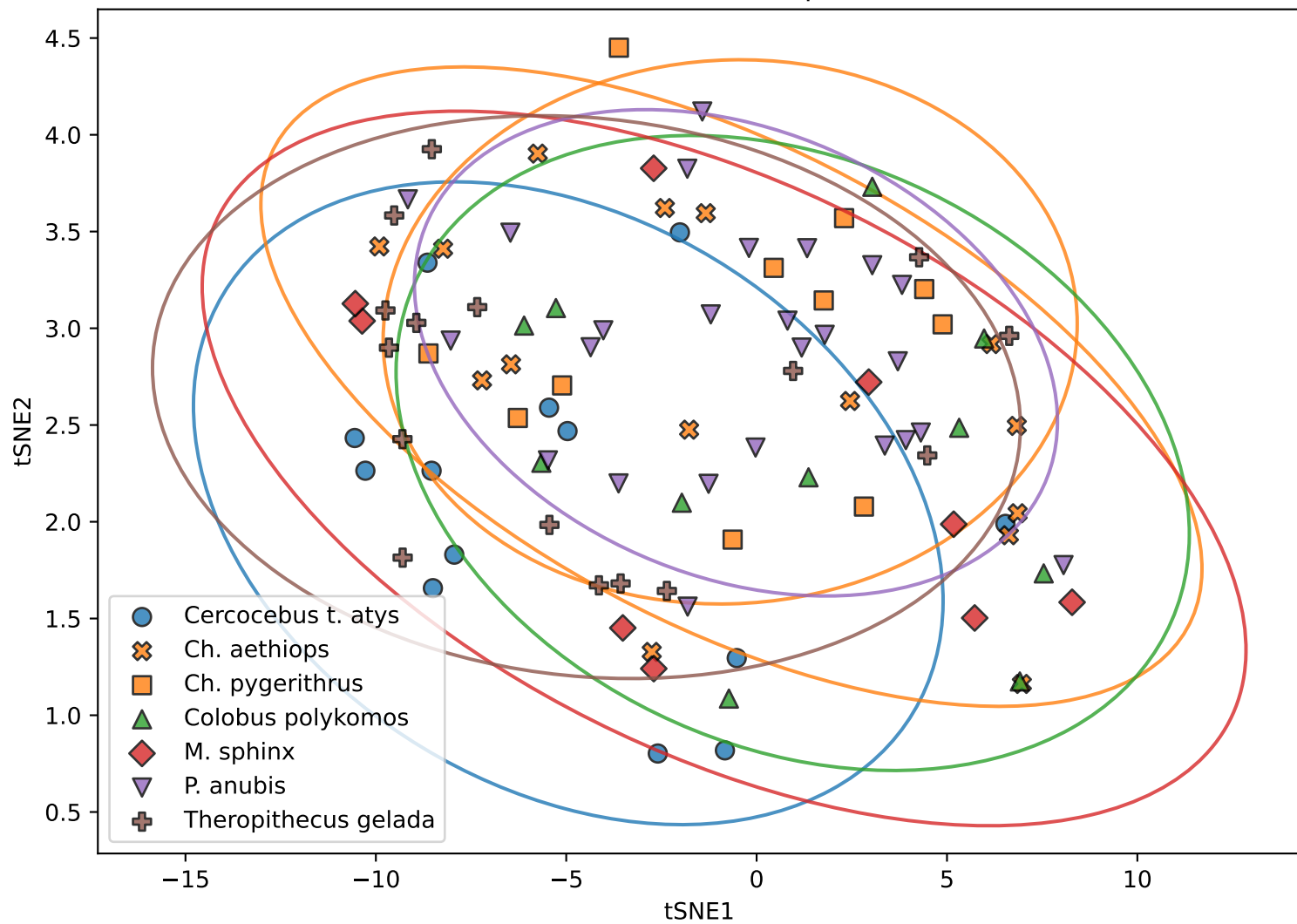

Supplement: Supplementary file 2 — Supplementary Information 2. [file 41598_2026_47350_MOESM2_ESM.zip › code_and_data_new/figures/reduced_dim_pdf/tsne_result_reduced_SPECIES _pca.pdf]

LDA Result

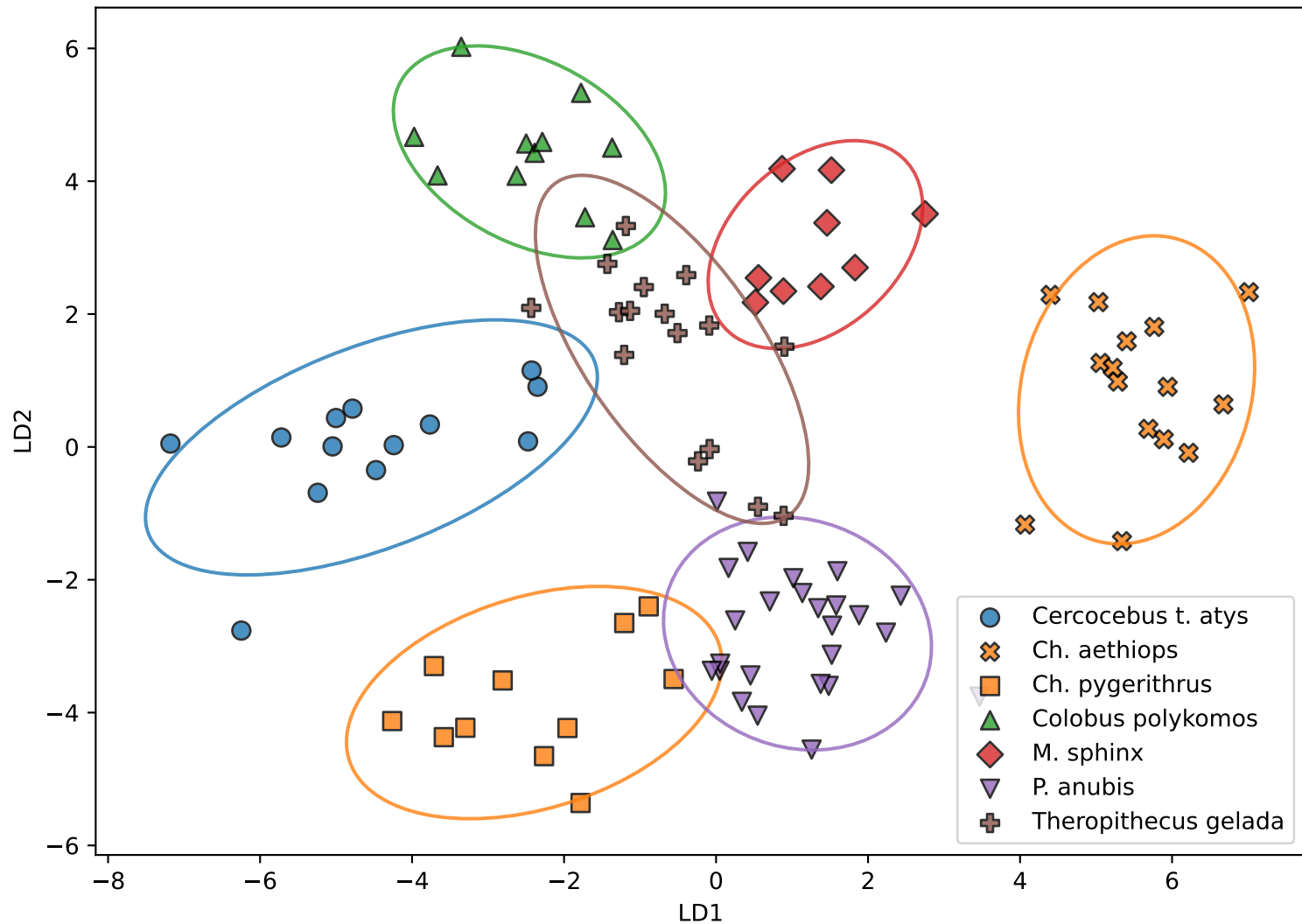

Supplement: Supplementary file 2 — Supplementary Information 2. [file 41598_2026_47350_MOESM2_ESM.zip › code_and_data_new/figures/reduced_dim_pdf/lda_result_SPECIES _lda.pdf]

UMAP on Reduced Components

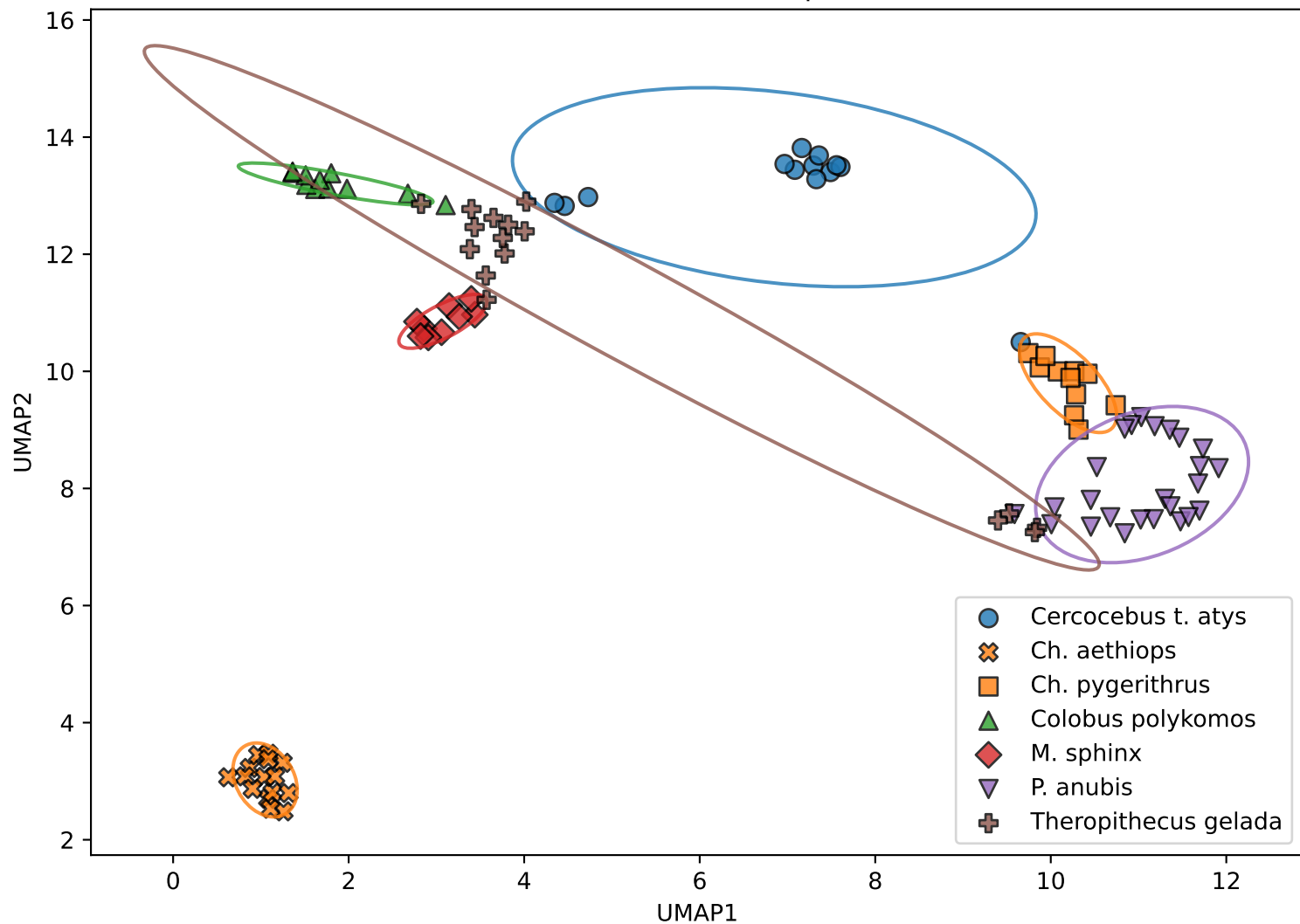

Supplement: Supplementary file 2 — Supplementary Information 2. [file 41598_2026_47350_MOESM2_ESM.zip › code_and_data_new/figures/reduced_dim_pdf/umap_result_reduced_SPECIES _lda.pdf]

t-SNE on Reduced Components

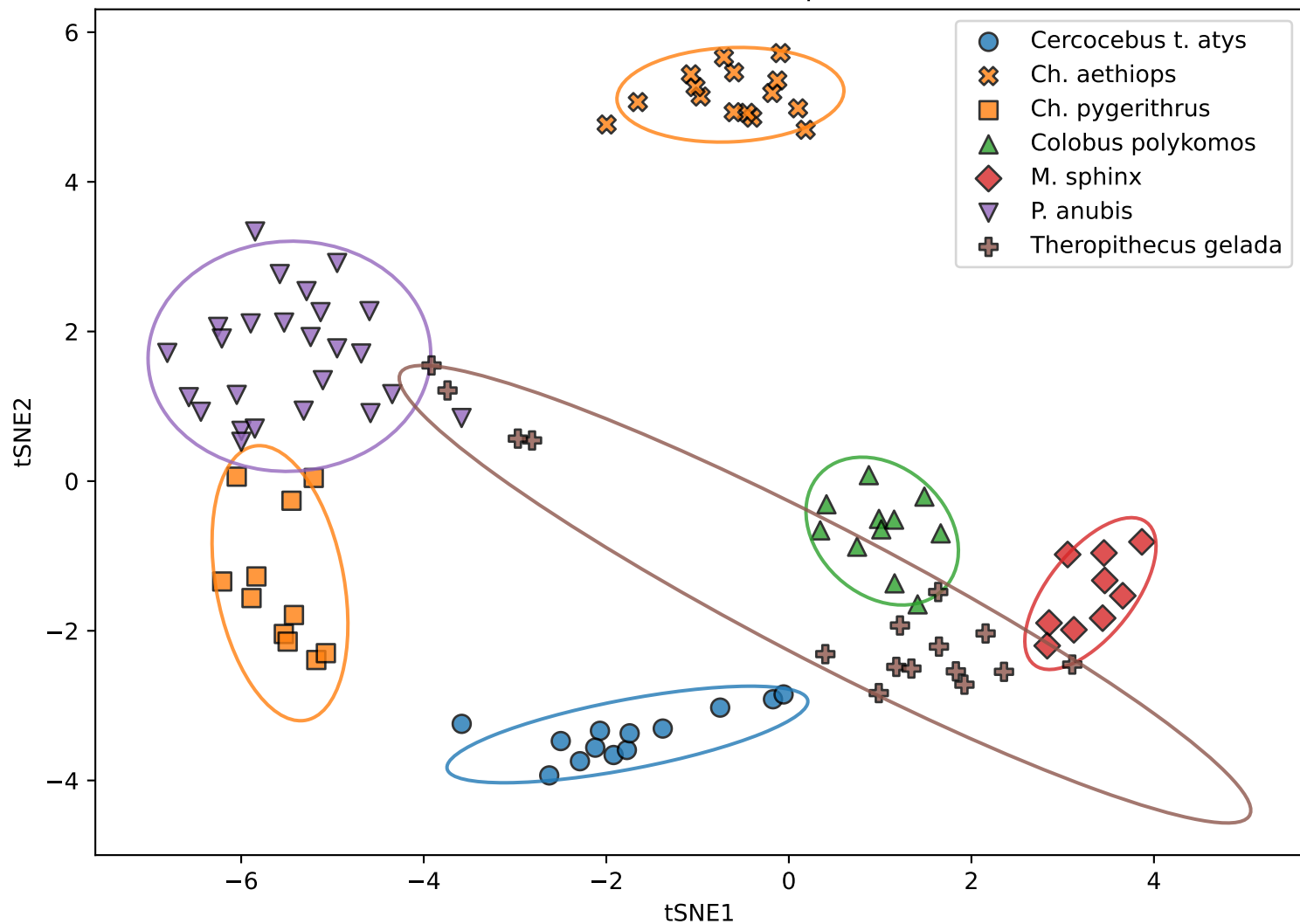

Supplement: Supplementary file 2 — Supplementary Information 2. [file 41598_2026_47350_MOESM2_ESM.zip › code_and_data_new/figures/reduced_dim_pdf/tsne_result_reduced_SPECIES _lda.pdf]

UMAP on Raw Data

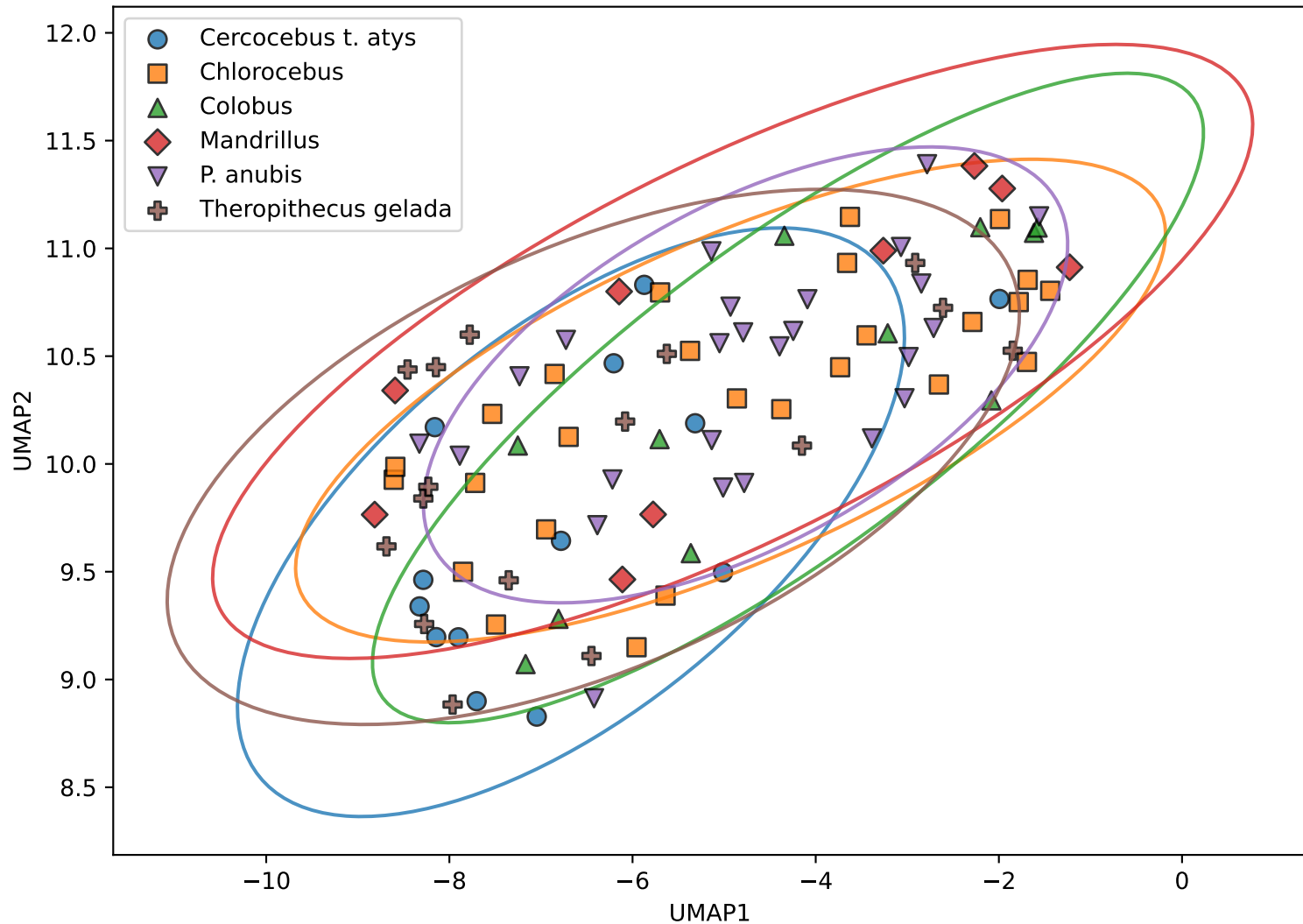

Supplement: Supplementary file 2 — Supplementary Information 2. [file 41598_2026_47350_MOESM2_ESM.zip › code_and_data_new/figures/raw_dim/umap_result_raw_GENNUS GROUP.pdf]

t-SNE on Raw Data

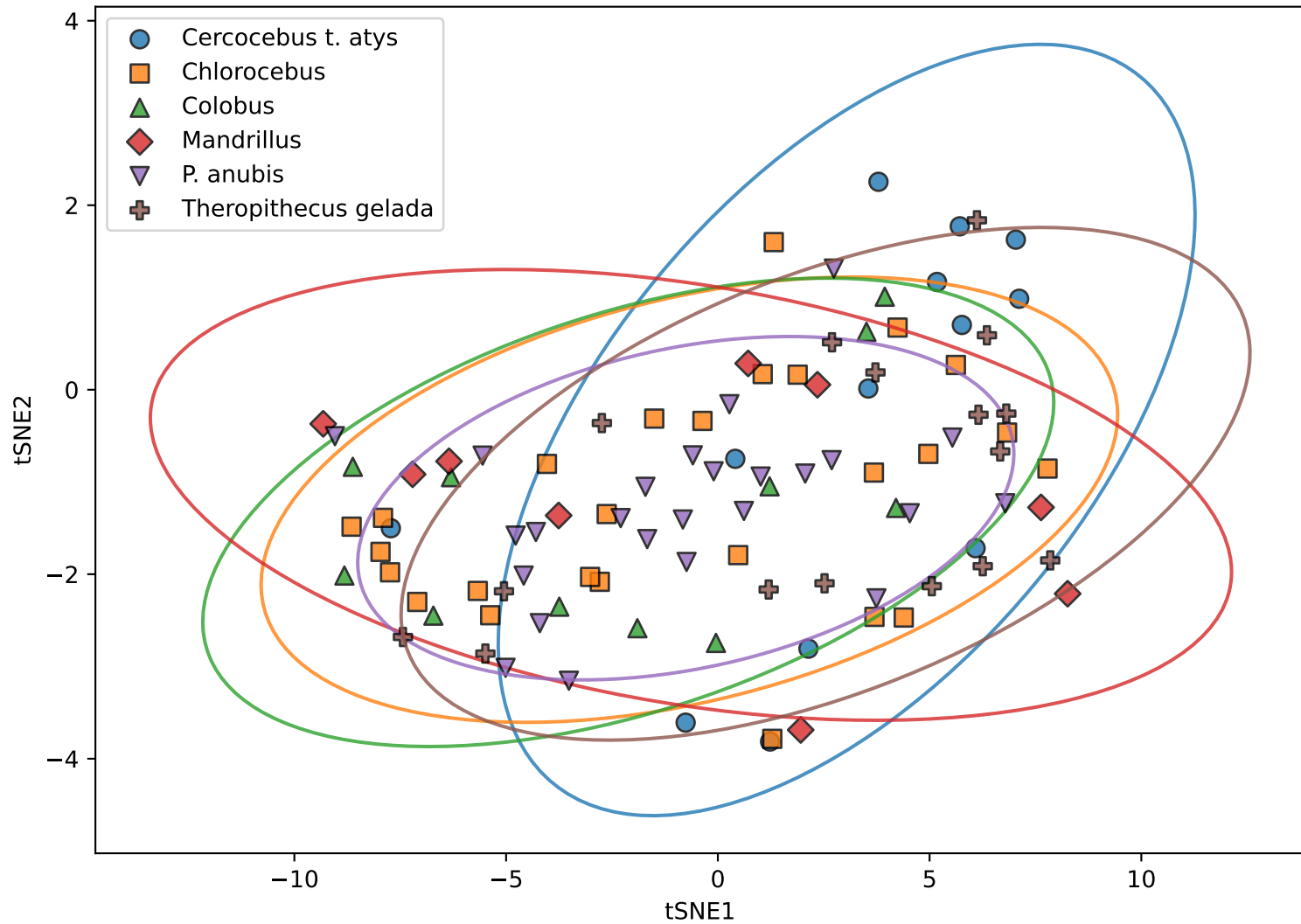

Supplement: Supplementary file 2 — Supplementary Information 2. [file 41598_2026_47350_MOESM2_ESM.zip › code_and_data_new/figures/raw_dim/tsne_result_raw_GENNUS GROUP.pdf]

UMAP on Raw Data

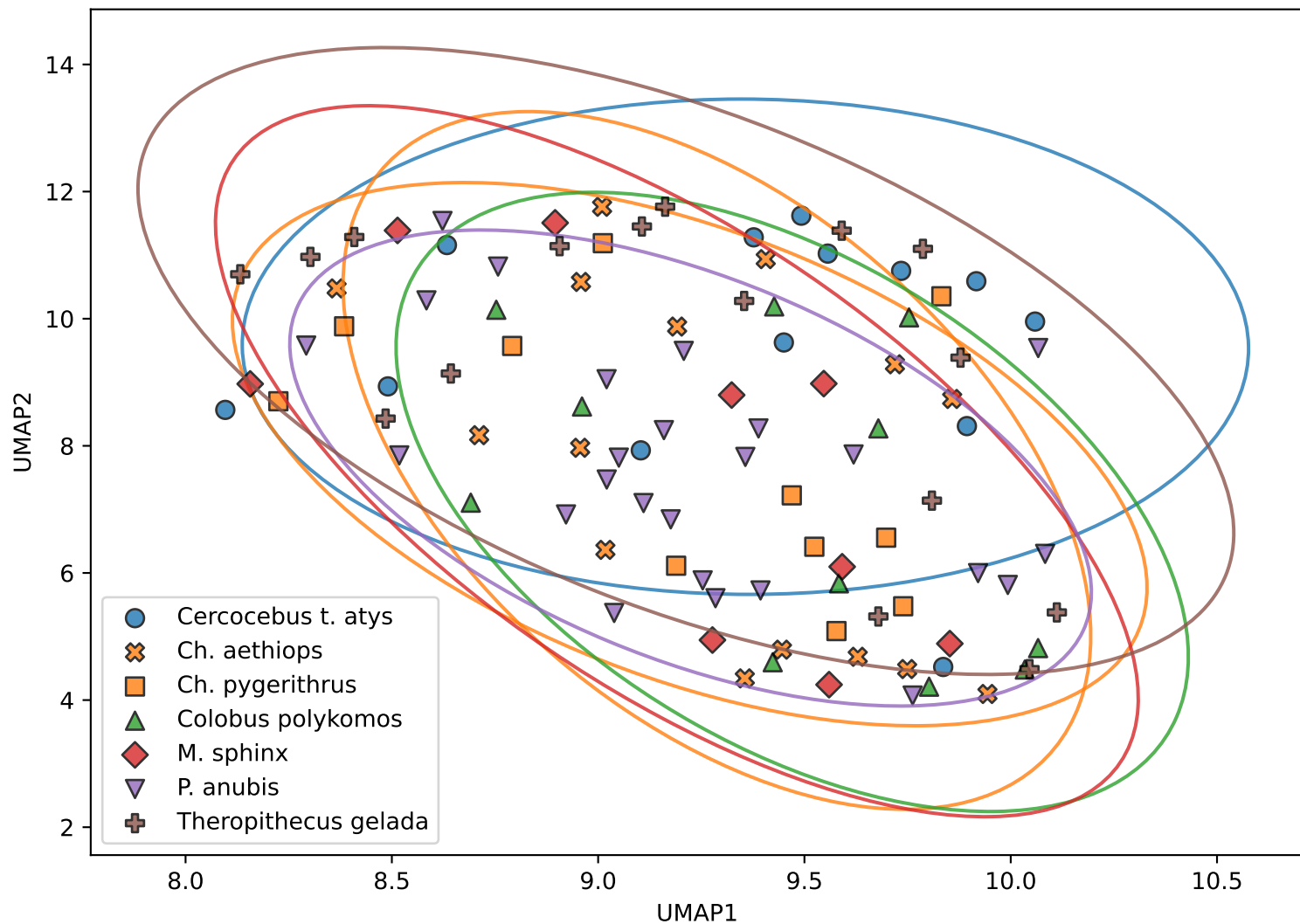

Supplement: Supplementary file 2 — Supplementary Information 2. [file 41598_2026_47350_MOESM2_ESM.zip › code_and_data_new/figures/raw_dim/umap_result_raw_SPECIES .pdf]

t-SNE on Raw Data

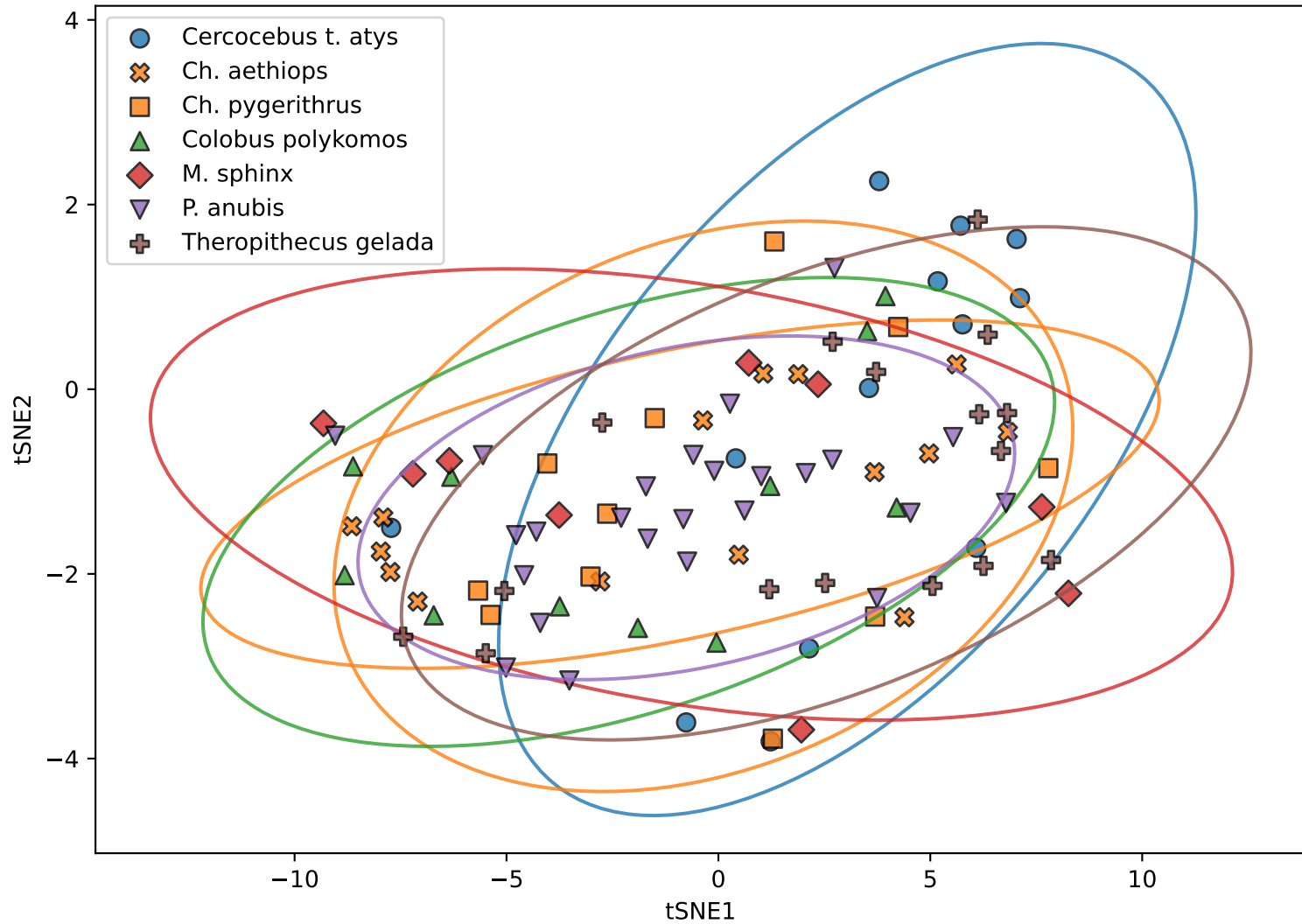

Supplement: Supplementary file 2 — Supplementary Information 2. [file 41598_2026_47350_MOESM2_ESM.zip › code_and_data_new/figures/raw_dim/tsne_result_raw_SPECIES .pdf]

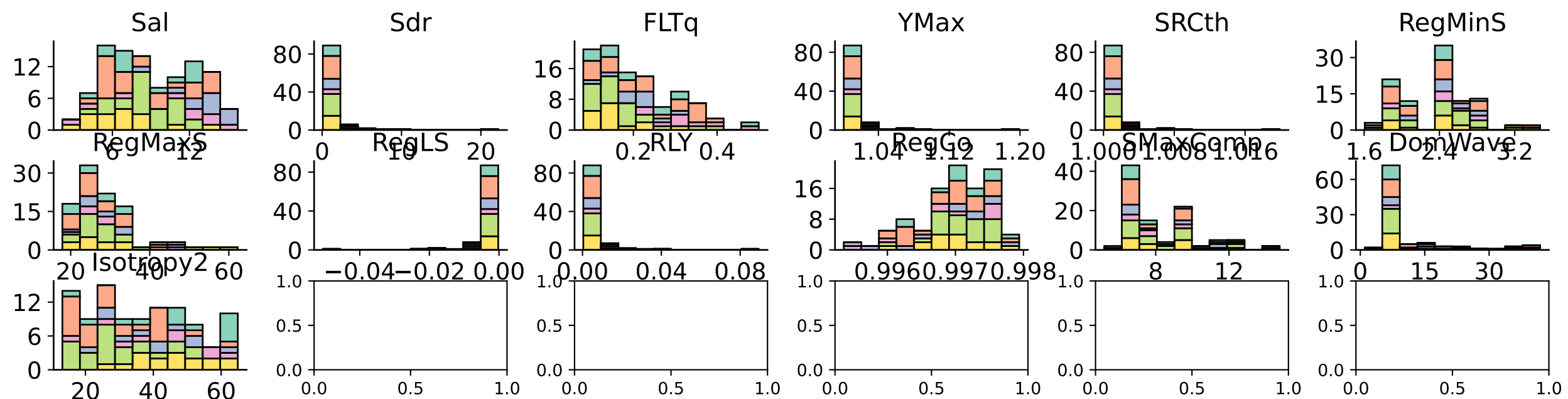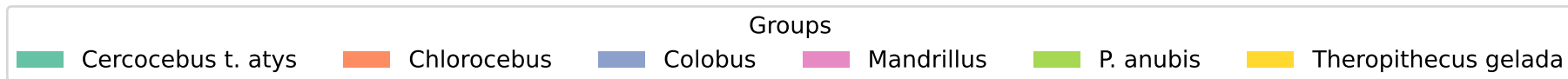

Supplement: Supplementary file 2 — Supplementary Information 2. [file 41598_2026_47350_MOESM2_ESM.zip › code_and_data_new/figures/non_normal_variables/All/grid_of_non_normal_variables.pdf]

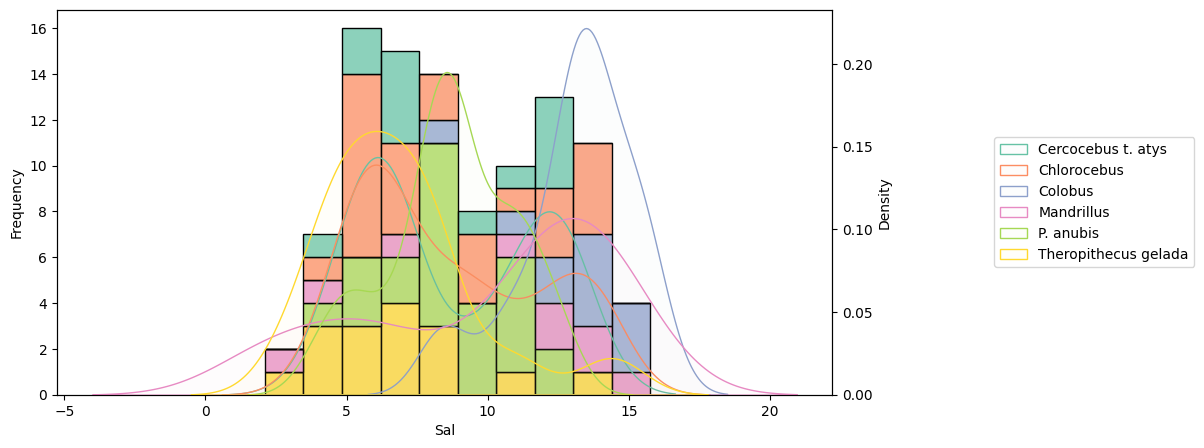

Supplement: Supplementary file 2 — Supplementary Information 2. [file 41598_2026_47350_MOESM2_ESM.zip › code_and_data_new/figures/non_normal_variables/iso/Sal.png]

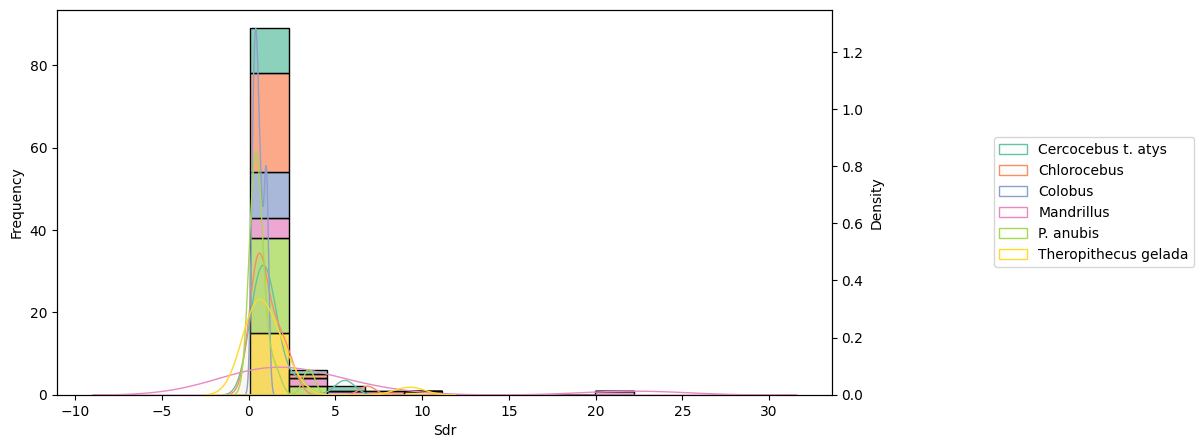

Supplement: Supplementary file 2 — Supplementary Information 2. [file 41598_2026_47350_MOESM2_ESM.zip › code_and_data_new/figures/non_normal_variables/iso/Sdr.png]

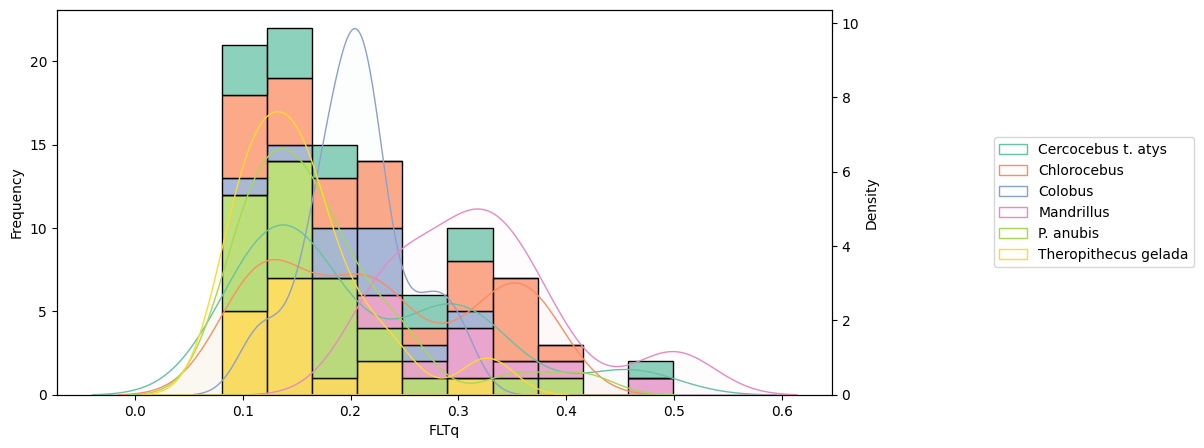

Supplement: Supplementary file 2 — Supplementary Information 2. [file 41598_2026_47350_MOESM2_ESM.zip › code_and_data_new/figures/non_normal_variables/iso/FLTq.png]

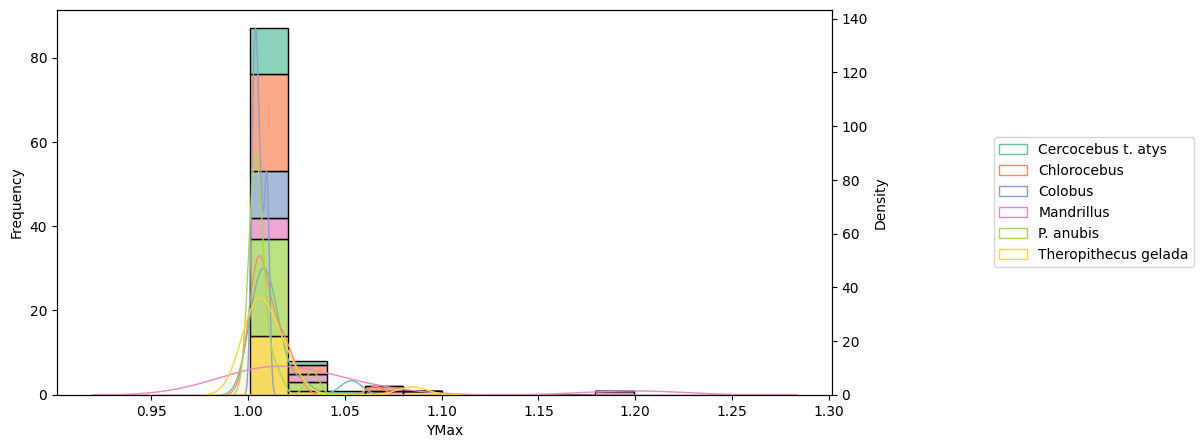

Supplement: Supplementary file 2 — Supplementary Information 2. [file 41598_2026_47350_MOESM2_ESM.zip › code_and_data_new/figures/non_normal_variables/ssfa/YMax.png]

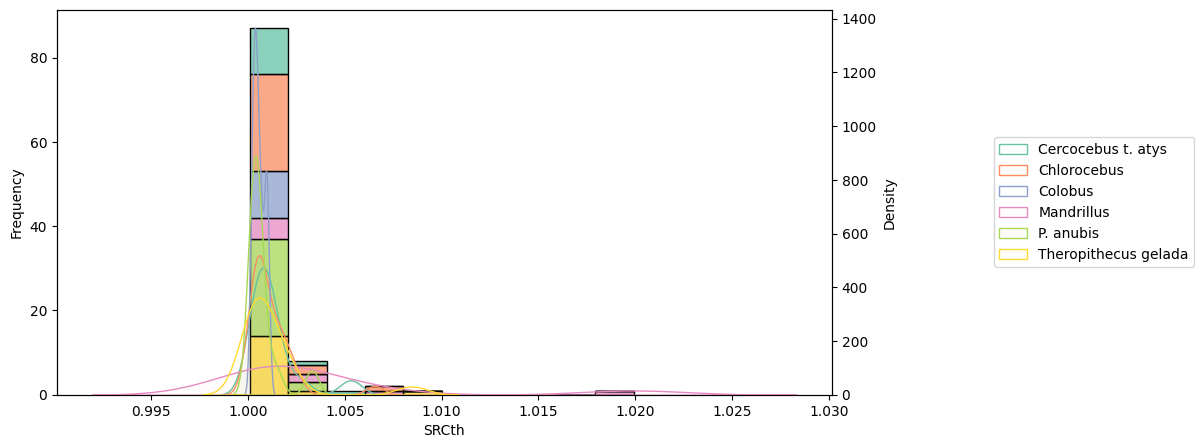

Supplement: Supplementary file 2 — Supplementary Information 2. [file 41598_2026_47350_MOESM2_ESM.zip › code_and_data_new/figures/non_normal_variables/ssfa/SRCth.png]

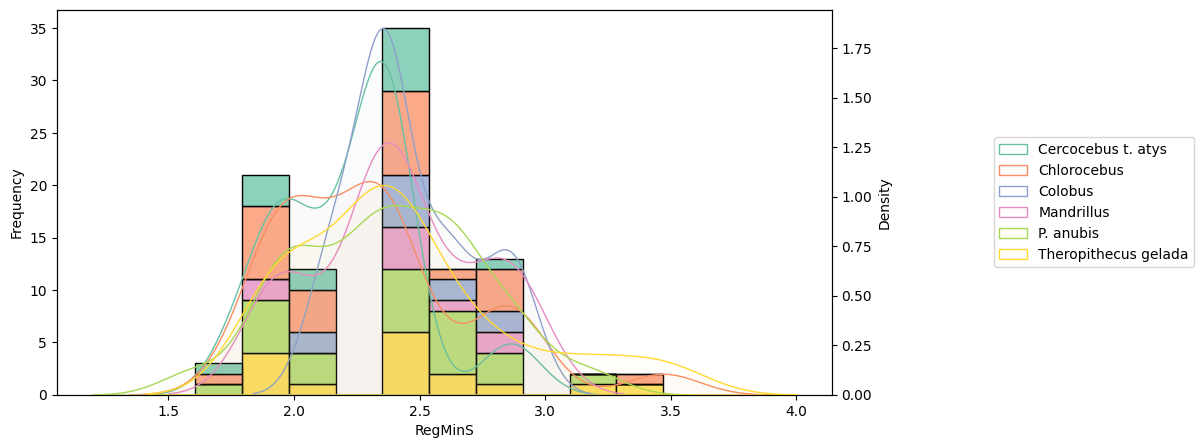

Supplement: Supplementary file 2 — Supplementary Information 2. [file 41598_2026_47350_MOESM2_ESM.zip › code_and_data_new/figures/non_normal_variables/ssfa/RegMinS.png]

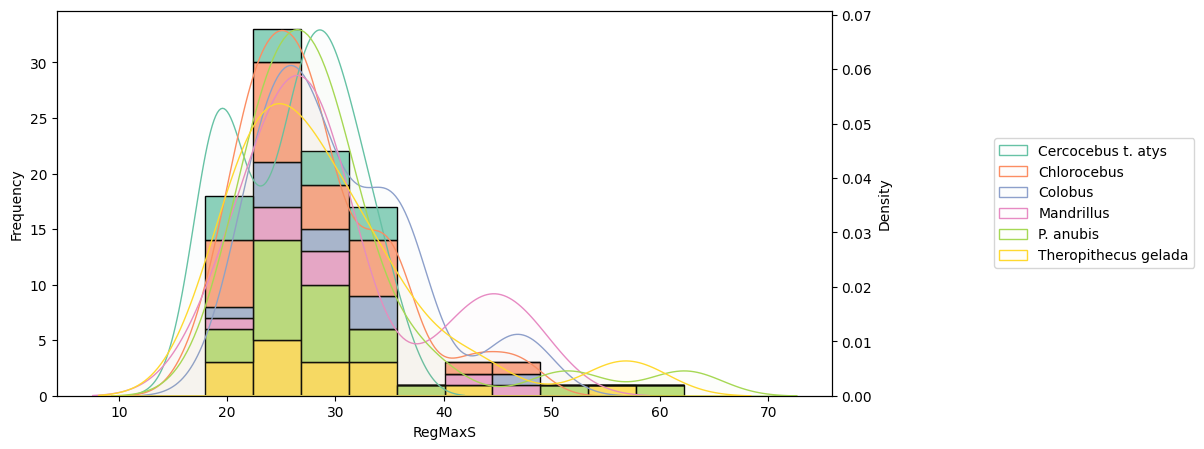

Supplement: Supplementary file 2 — Supplementary Information 2. [file 41598_2026_47350_MOESM2_ESM.zip › code_and_data_new/figures/non_normal_variables/ssfa/RegMaxS.png]

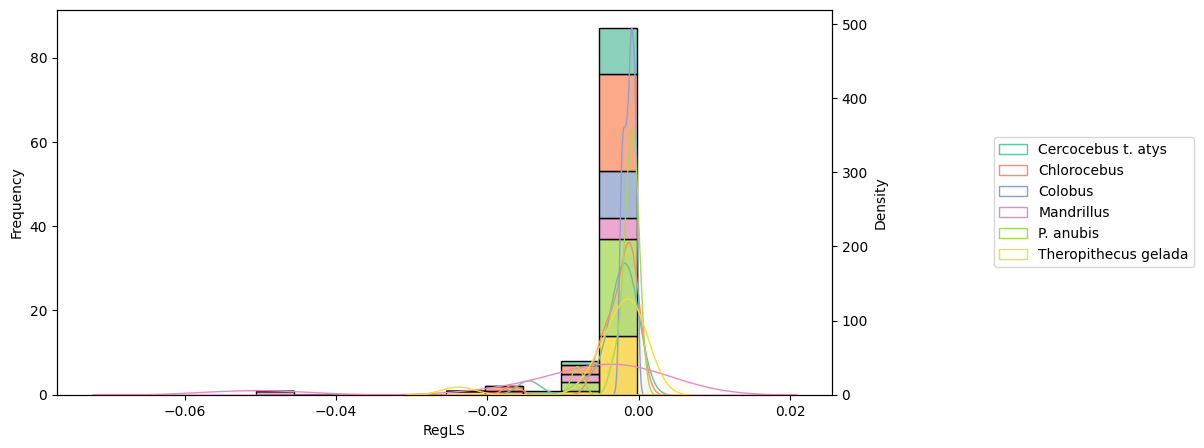

Supplement: Supplementary file 2 — Supplementary Information 2. [file 41598_2026_47350_MOESM2_ESM.zip › code_and_data_new/figures/non_normal_variables/ssfa/RegLS.png]

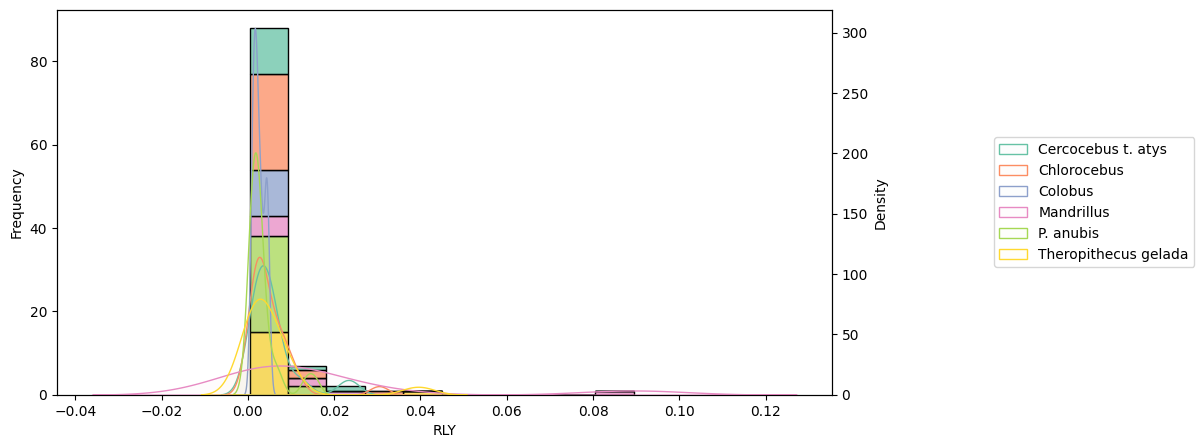

Supplement: Supplementary file 2 — Supplementary Information 2. [file 41598_2026_47350_MOESM2_ESM.zip › code_and_data_new/figures/non_normal_variables/ssfa/RLY.png]

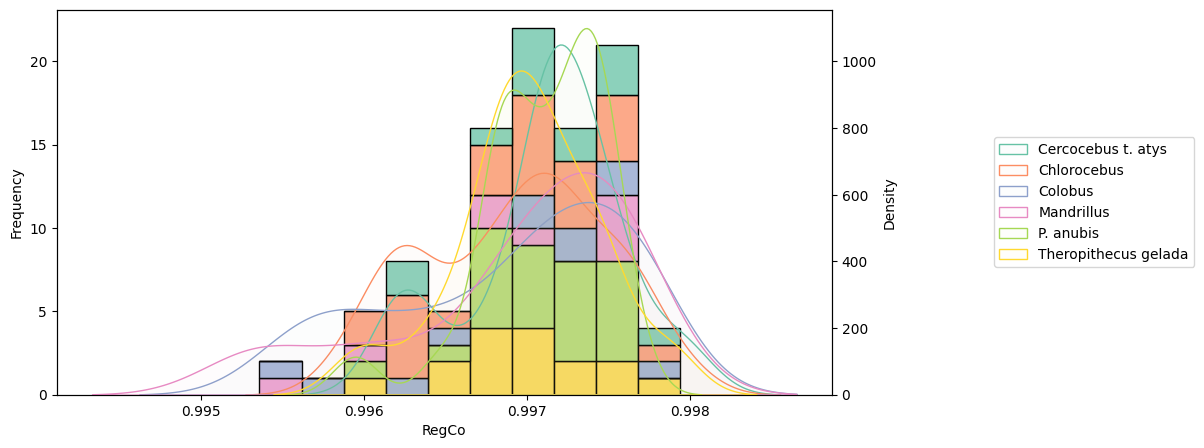

Supplement: Supplementary file 2 — Supplementary Information 2. [file 41598_2026_47350_MOESM2_ESM.zip › code_and_data_new/figures/non_normal_variables/ssfa/RegCo.png]

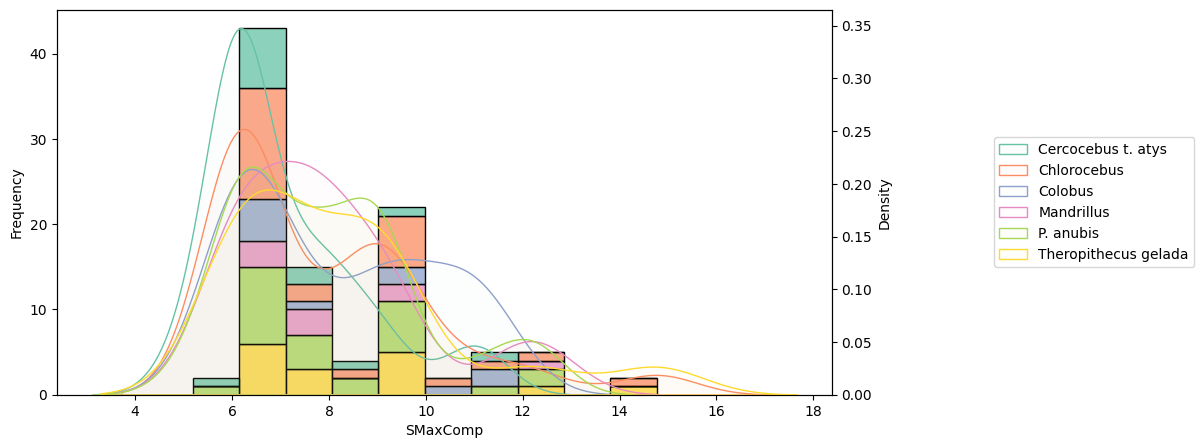

Supplement: Supplementary file 2 — Supplementary Information 2. [file 41598_2026_47350_MOESM2_ESM.zip › code_and_data_new/figures/non_normal_variables/ssfa/SMaxComp.png]

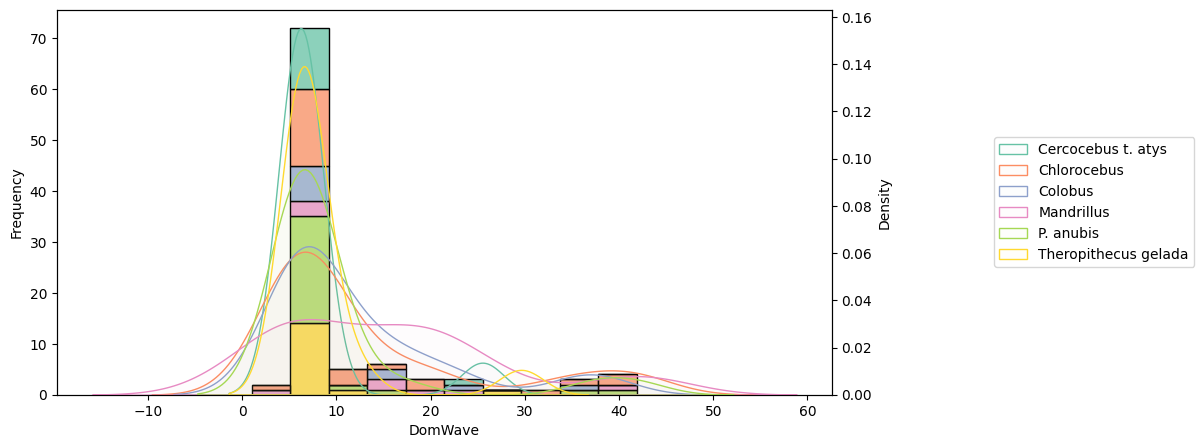

Supplement: Supplementary file 2 — Supplementary Information 2. [file 41598_2026_47350_MOESM2_ESM.zip › code_and_data_new/figures/non_normal_variables/extra/DomWave.png]

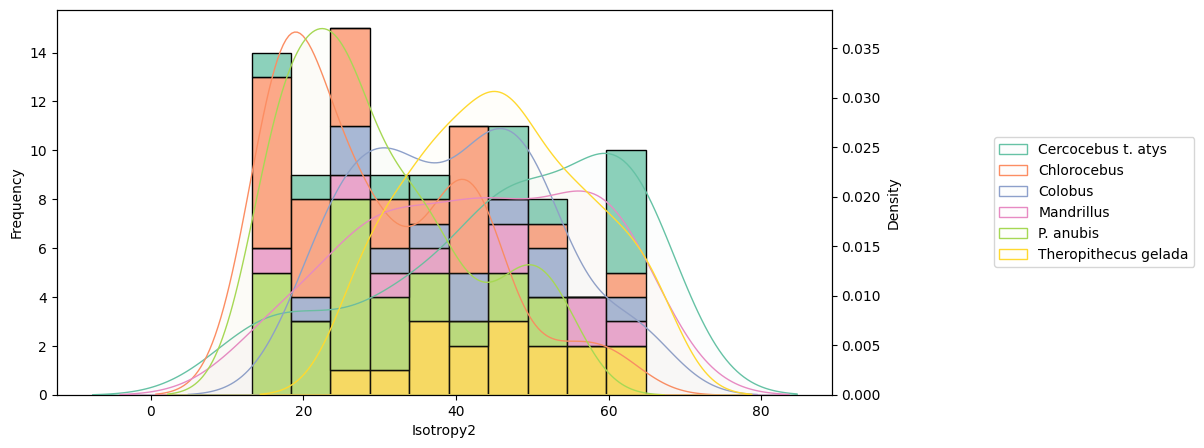

Supplement: Supplementary file 2 — Supplementary Information 2. [file 41598_2026_47350_MOESM2_ESM.zip › code_and_data_new/figures/non_normal_variables/extra/Isotropy2.png]
